# Hairless Streaks in Cattle Implicate TSR2 in Early Hair Follicle Formation

**DOI:** 10.1371/journal.pgen.1005427

**Published:** 2015-07-23

**Authors:** Leonardo Murgiano, Vera Shirokova, Monika Maria Welle, Vidhya Jagannathan, Philippe Plattet, Anna Oevermann, Aldona Pienkowska-Schelling, Daniele Gallo, Arcangelo Gentile, Marja Mikkola, Cord Drögemüller

**Affiliations:** 1 Institute of Genetics, Vetsuisse Faculty, University of Bern, Bern, Switzerland; 2 DermFocus, University of Bern, Bern, Switzerland; 3 Developmental Biology Program, Institute of Biotechnology, University of Helsinki, Helsinki, Finland; 4 Institute of Animal Pathology, Vetsuisse Faculty, University of Bern, Bern, Switzerland; 5 Division of Neurological Sciences, DCR-VPH, Vetsuisse Faculty, University of Bern, Bern, Switzerland; 6 Clinic for Reproductive Medicine, Vetsuisse Faculty, University of Zurich, Zurich, Switzerland; 7 Department of Veterinary Medical Sciences, University of Bologna, Ozzano dell’Emilia, Italy; Biosciences Research Division, Department of Primary Industries, AUSTRALIA

## Abstract

Four related cows showed hairless streaks on various parts of the body with no correlation to the pigmentation pattern. The stripes occurred in a consistent pattern resembling the lines of Blaschko. The non-syndromic hairlessness phenotype observed occurred across three generations of a single family and was compatible with an X-linked mode of inheritance. Linkage analysis and subsequent whole genome sequencing of one affected female identified two perfectly associated non-synonymous sequence variants in the critical interval on bovine chromosome X. Both variants occurred in complete linkage disequilibrium and were absent in more than 3900 controls. An *ERCC6L* missense mutation was predicted to cause an amino acid substitution of a non-conserved residue. Analysis in mice showed no specific *Ercc6l* expression pattern related to hair follicle development and therefore *ERCC6L* was not considered as causative gene. A point mutation at the 5'-splice junction of exon 5 of the *TSR2*, *20S rRNA accumulation*, *homolog (S*. *cerevisiae)*, gene led to the production of two mutant transcripts, both of which contain a frameshift and generate a premature stop codon predicted to truncate approximately 25% of the protein. Interestingly, in addition to the presence of both physiological *TSR2* transcripts, the two mutant transcripts were predominantly detected in the hairless skin of the affected cows. Immunohistochemistry, using an antibody against the N-terminal part of the bovine protein demonstrated the specific expression of the TSR2 protein in the skin and the hair of the affected and the control cows as well as in bovine fetal skin and hair. The RNA hybridization *in situ* showed that *Tsr2* was expressed in pre- and post-natal phases of hair follicle development in mice. Mammalian TSR2 proteins are highly conserved and are known to be broadly expressed, but their precise *in vivo* functions are poorly understood. Thus, by dissecting a naturally occurring mutation in a domestic animal species, we identified TSR2 as a regulator of hair follicle development.

## Author Summary

The identification of causal mutations of rare monogenic disorders provides an insight into the function of single genes. We herein report an example which demonstrates that the bovine species presents an excellent system for identifying these inherited phenotypes. The individual health status of modern dairy cows is well monitored, and emerging disorders are routinely recorded. An Italian breeder of ~500 Pezzata Rossa cattle reported a case of congenital streaked hairlessness. Three additional, closely related cows, showing similar hairless pattern following Blaschko’s lines were subsequently observed. A causative mutation was discovered in a previously uncharacterized rRNA processing gene. Cows possessing a single copy of this *TSR2* mutation located on the X chromosome showed a mosaic skin pattern which is very likely due to the skewed inactivation of the X-chromosome, also known as lyonization. The expression of TSR2 was shown in skin and hair of cattle and mice. This study is the first to implicate an essential role for TSR*2* during hair follicle development and reflects once more the potential of using rare diseases in cows to gain additional insights into mammalian biology.

## Introduction

In 1901, the German dermatologist Alfred Blaschko proposed that congenital linear skin lesions could develop independently of the nervous system [1]. Blaschko observed a common non-random developmental pattern of the skin and described it extensively depicting the shape of the pattern lines [1, 2]. The so-called lines of Blaschko run along the sides of the individual’s body, bending in a roughly S-shaped pattern toward the ventral part, forming a typical symmetrical V shape near the center of the back [3]. These lines become clinically manifest in the heterozygous state of various human X-linked inherited defects, such as incontinentia pigmenti, focal dermal hypoplasia, chondrodysplasia punctata, hypohidrotic ectodermal dysplasia, and Menkes syndrome [4, 5, 6]. The inactivation of one X chromosome (XCI), which leads to mosaicism for cells with the mutant allele silenced, can explain different patterns of functional mosaicism in over a dozen X-linked conditions [5, 6]. The pattern of cutaneous mosaicism can be tracked back to the type of cell affected, and its trajectory of migration and proliferation during embryogenesis [3, 4]. Lines of Blaschko are due to ectodermal precursor cells which migrate and proliferate along these tracts. In female mammalian embryos, one of the two X chromosomes in each somatic cell is silenced in early development, albeit additional events can skew the inactivation [7]. Consequently, every female is a functional mosaic of cells, each exclusively expressing her maternal or paternal copy of X-chromosomal genes. In general, the effects of an X-linked gene mutation depend on XCI patterns. For genes subject to XCI, a mutation which affects males does not necessarily affect females who can be unaffected either due to random XCI or by selective skewing in favor of cells which express the normal allele [7].

Several forms of inherited alopecia have been described in domestic animal species (OMIA 001702–9913, OMIA 001702–9615, OMIA 001702–9796, OMIA 001702–9685, OMIA 001702–9825, OMIA 000031–9615, OMIA 000030–9685, OMIA 000030–9031, OMIA 000030–9940) [8], including hairlessness and X-linked phenotypes (OMIA 000543–9913) [9, 10, 11]. Our group has recently reported a family of horses in which females developed signs of a skin disorder reminiscent of human incontinentia pigmenti (OMIA 001899–9796) [10]. Notably, the affected horses showed congenital streaks of varying coat color which followed the lines of Blaschko, and a causative nonsense mutation was found in the X-chromosomal *IKBKG* gene [10]. In general, the dissection of naturally occurring spontaneous mutations in domestic animals can lead to important insights into developmental genetics, as has been shown for hairless dogs carrying a *FOXI3* mutation (OMIA 000323–9615) [12].

In a dairy farm in Friuli (Italy), an X-linked inherited non-syndromic congenital hairlessness phenotype was detected in four cows showing hairless stripes in a consistent pattern resembling the lines of Blaschko. The condition was strikingly similar to the so-called streaked hairlessness phenotype reported 60 years ago in female Holstein cattle in North America, which was supposed to be X-linked dominant inherited with a lethal effect on hemizygous male embryos (OMIA 000542–9913) [11]. The goal of the present study was to identify the causative gene for bovine streaked hairlessness using a positional cloning strategy.

## Results

### Matrilineal streaked hairlessness

**Fig 1:**
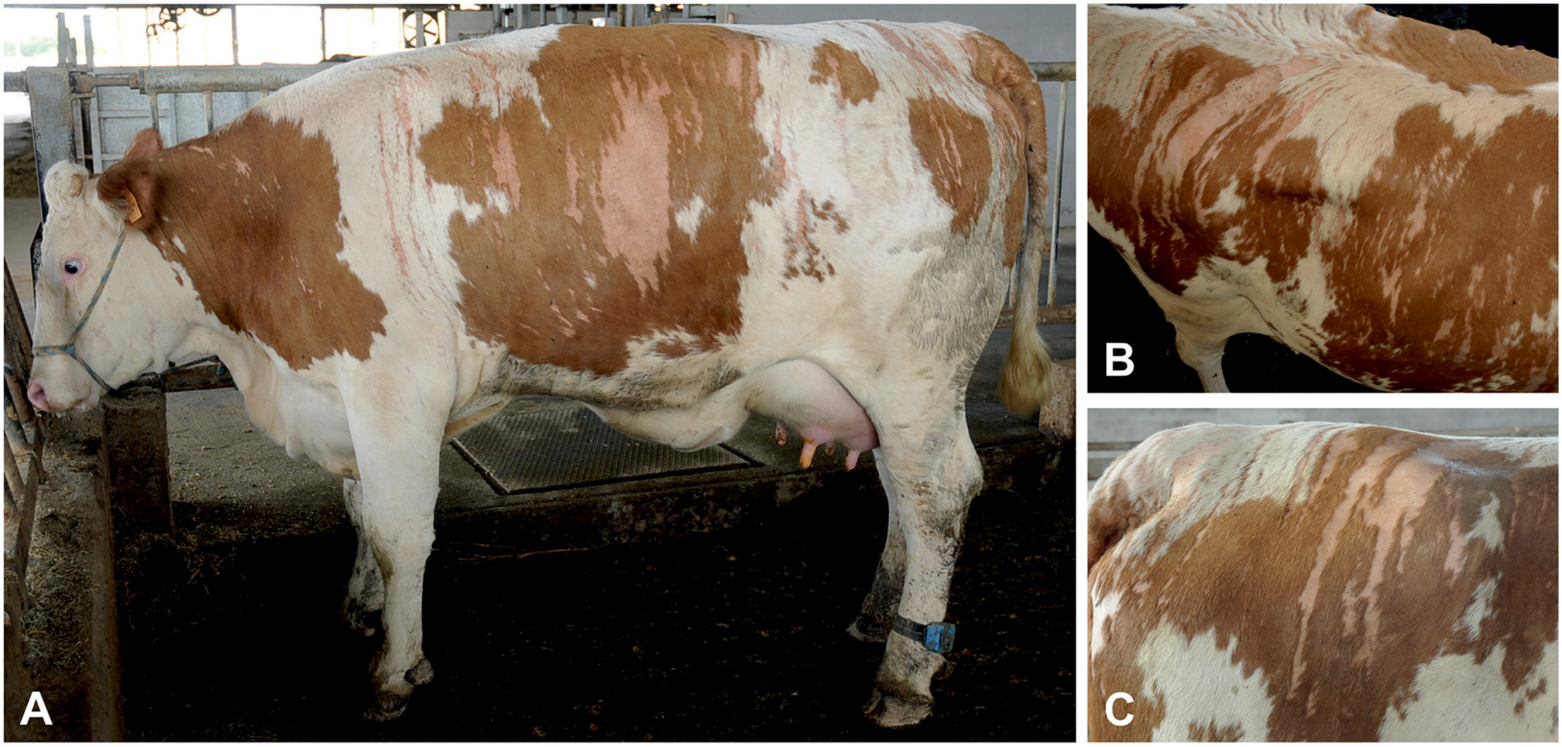
Streaked hairlessness in Pezzata Rossa cattle. (A) The sharply demarcated hairless areas are clearly distinguishable. (B) The V-shaped pattern on the back is illustrated. (C) Note that the hairless pattern is unrelated to coat color. The animal shown corresponds to case 1 in Fig 2A.

The presence of skin lesions was detected in a total of four related female Pezzata Rossa cattle. The hairless lesions, present from birth, varied in their extent and size in the different animals but were all characterized by streaks of hairless areas following a vertical pattern. At the time of the first consultation the most severely affected animal was a 21-month-old pregnant heifer (case 1). Hairless streaks were present bilaterally along both sides of the animal (Fig 1A and S1 Fig). Their V-shaped symmetrical convergence at the level of the back resulted in a fishbone-like pattern (Fig 1B and 1C). On the right flank, approximately over the last three ribs, a larger area of hairlessness was also present (Fig 1A). Hairless streaks were also present on the head. The skin of the udder presented diffuse non-streaked hypotrichosis. The lesions occurred without any association to the coat color, both pigmented and unpigmented areas being affected (Fig 1C). Apart from the hairlessness, the skin of the affected areas was smooth, of normal color and without any crusts. No macroscopic intermediate aspect was present between the affected area and the surrounding skin. No abnormal cutaneous pain sensations by pressure, pricking or pinching stimuli, were observed at the level of the hairless areas as compared to the haired skin. Pruritus was also not apparent. The heifer showed no other clinical findings. The lesions remained practically unchanged during the three-year observation period, and no sign of hair regrowth was observed. During this period, the animal gave birth to three healthy calves, two males and one female. Both males were sold at the age of approximately one month and did not show any signs of hairlessness at that time. The female offspring of case 1 was examined for the last time at the age of 16 months and no skin abnormalities were detected, although similar, but less severe streaked hairlessness, was present in the dam (case 2) and in the granddam (case 3) of the aforementioned heifer (case 1). In the mother, the streaked lesions were limited to the rump and shoulders (S1 Fig), whereas, in the grandmother, the phenotype was diffusely evident at the level of the rump, back and hips (S1 Fig). The reported lesions had been present since birth and had the same characteristics as those described above (S1 Fig). Streaked hairless lesions were also present at the level of the rump, shoulders and the dorsal portion of the ribs of a forth case, a 15-month-old heifer (case 4), a half-sibling of case 2 on the side of their dam (S1 Fig). No other clinical signs were observed in these three additional cases. No alteration in the production of milk was reported but, with respect to the cows’ fertility, the owner reported that case 3 failed to conceive for five successive inseminations.

**Fig 2:**
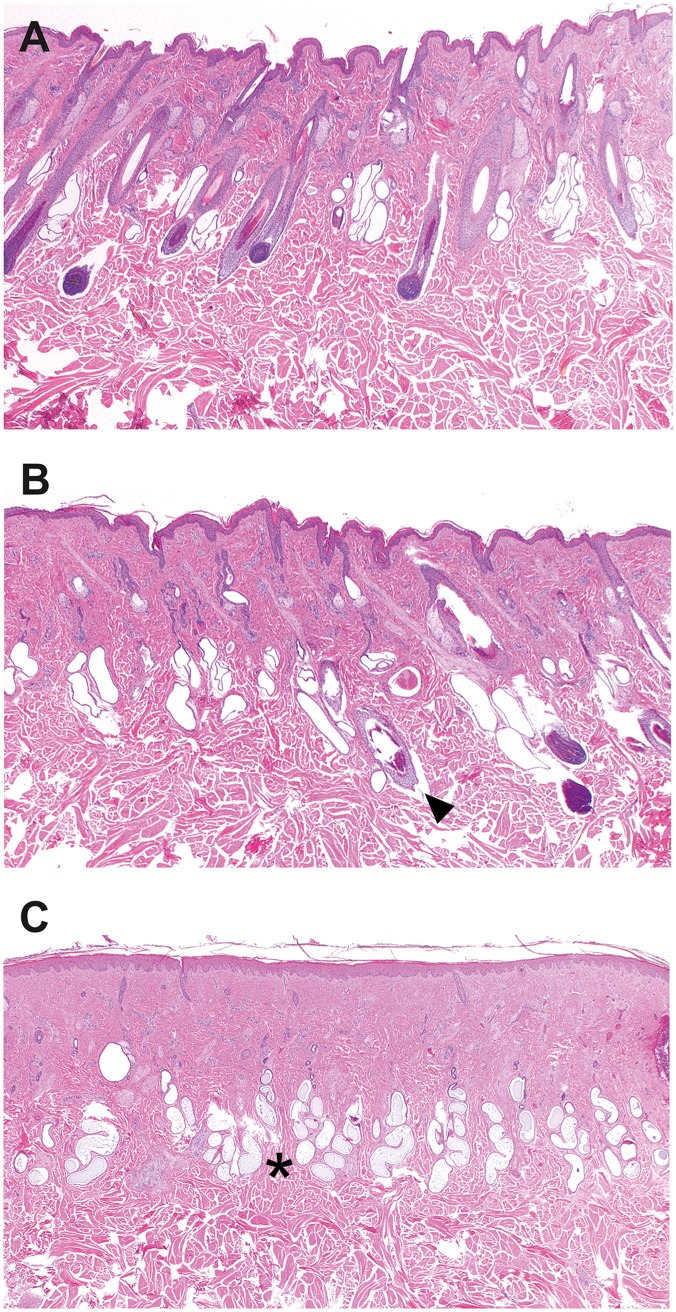
Histopathology of skin samples from a cow with streaked hairlessness. (A) Haired skin of an affected cow (case 1 in Fig 2A), showing no abnormalities in hair follicle size and distribution. (B) Border between the haired and the hairless skin. Note the presence of normal and abnormal hair follicles. Hair follicles of normal size with bulbs reaching into the subcutis were adjacent to dysplastic follicles characterized by a distorted contour and a smaller diameter (arrow). Sebaceous glands are present. (C) Loss of normal hair follicles and sebaceous glands in the hairless skin whereas the sweat glands (asterisk) are all present. Haematoxylin and eosin staining, magnifications 20X.

The hair follicles in the biopsies from the haired skin were normally distributed, and size and shape were comparable with hair follicles in skin biopsies from non-affected cows (Fig 2A). In the skin biopsies from the hairless sites, the vast majority of the hair follicles and sebaceous glands were missing whereas the sweat glands, their ducts and the arrector pili muscles were present (Fig 2C). Dysplastic or miniaturized hair bulbs or remnant fibrous sheaths surrounding the bulb were occasionally present. In addition, remnants of infundibula were rarely seen. In the biopsies from the haired-hairless border, a mixture of normal hair follicles and dysplastic infundibuli were present (Fig 2C). The dysplastic infundibula were smaller than those of normal hair follicles, had an irregular outer contour and were often associated with the sebaceous glands and the ducts of the sweat glands (S2 Fig).

**Fig 3:**
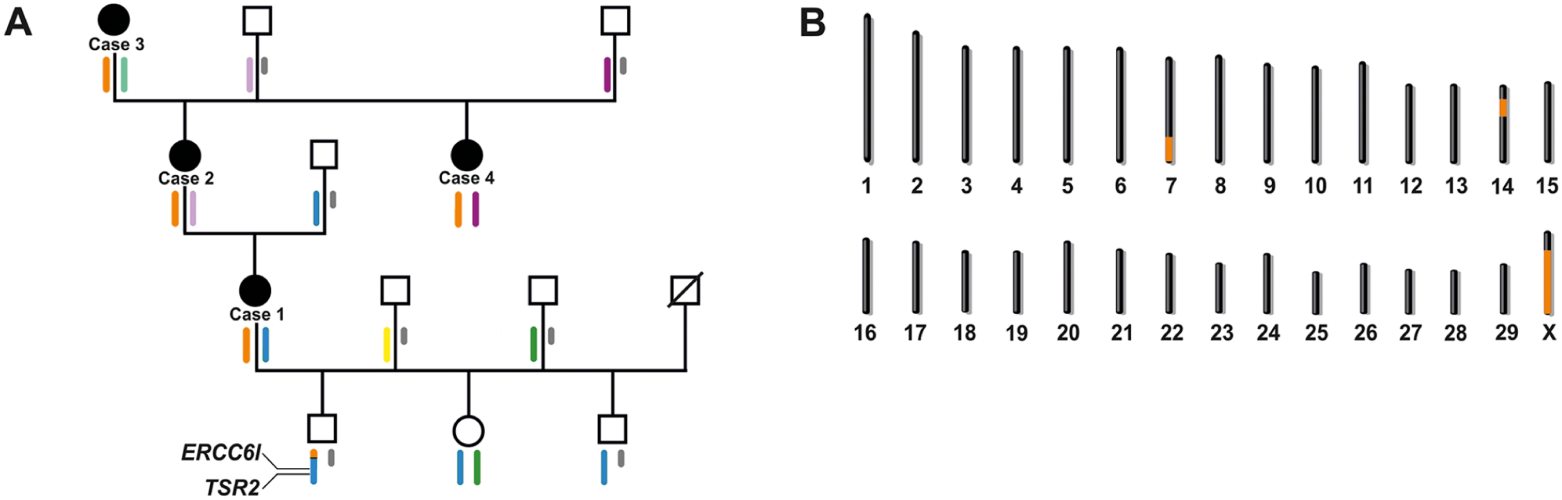
X-linked inheritance of bovine streaked hairlessness. (A) Family tree of four affected females (black symbols). The males are indicated by squares, the females by circles. Deduced X chromosome haplotypes are shown colored below the individuals. Y chromosomes are shown in grey. Note the red haplotype spanning the entire chromosome X is shared by all the affected animals. In one of the two non-affected male offspring of case 1, a recombinant haplotype was detected which helped to exclude the proximal part of the X chromosome. The position of both genes (*TSR2* and *ERCC6L*) containing disease-associated variants are shown. (B) Genome regions showing positive LOD scores in linkage analysis are shown in orange.

The matrilinear descent of the affected animals, two of them being the female offspring of the oldest one and another being her granddaughter, is depicted in Fig 3A. Each one of the offspring was generated using different artificial insemination sires without any other comparably common ancestor of the four affected animals. Taken together, the segregation pattern of the observed phenotype can be explained by a monogenic X-linked inherited mutation causing the streaked hairlessness condition.

### Linkage mapping supports X-linked inheritance

Karyotype analysis of three of the affected animals (cases 1, 2 and 3) and one healthy male offspring of case 1 was initially performed. The karyotypes appeared completely normal revealing no evidence for any visible numerical or gross structural chromosomal aberration (S3 Fig). To map candidate regions for the streaked hairlessness condition, we genotyped four affected cows, and a total of eight available normal family members for 777,962 SNPs (Fig 3A). A haplotype analysis searching for disease-linked haplotypes shared across the four affected animals was carried out. A 29.2 Mb shared haplotype on BTA 7 (position 82,876,246 to the end of the chromosome) and an 11.5 Mb shared haplotype on BTA 14 (position 21,284,128 to 32,783,095) were found. All four affected cows shared one single haplotype spanning the entire X chromosome (Fig 3A). In addition, all three non-affected offspring of case 1 were checked for the presence of the shared X haplotype and a recombinant X chromosome was detected in one son. A multipoint parametric linkage analysis revealing positive LOD scores on bovine chromosomes (BTA) 7, 14 and X was carried out (Fig 3B and S4 Fig). In a critical interval of 118.1 Mb on chromosome X (position 30,947,683 down to the end of the chromosome) the highest multipoint LOD score of 1.405 was detected (Fig 3B and S4 Fig).

### Two associated coding variants on chromosome X

The entire genome of one affected cow (case 1) was sequenced and the three genomic regions showing positive LOD scores in the linkage analysis were then focused on. Since the phenotype was mild and did not affect normal life, all variants present in the mapped regions including synonymous, nonsense and missense exon variants, and variants in the introns and splicing sites of annotated genes and intergenic polymorphisms were considered as potential causative mutations.

A total of about 8.8 million including 86,326 coding variants were called with respect to the reference genome (Table 1). A comparison was then made between all 361,134 DNA variants in the candidate regions present in the sequenced affected cow and 83 cow genomes of various cattle breeds which had been sequenced in our laboratory in the course of other studies. Thanks to this first step of filtering, the number of variants was reduced to 2593 including 21 coding variants of which all but one present on chromosome X. In a subsequent step, our membership in the 1000 bull genomes project was made use of [13] and the run4 variant database including 1119 genomes was used. This second filter step allowed the exclusion of 2564 variants remaining with 29 private sequence variants: 27 private variants located in intergenic and intronic regions on BTA 7 and two private non-synonymous coding variants located on the X chromosome in the *excision repair cross-complementation group 6-like* (*ERCC6L*) and *TSR2*, *20S rRNA accumulation*, *homolog (S*. *cerevisiae)* (*TSR2*) genes (Table 1, S1 Table). Due to the observed segregation pattern in the affected cattle family and the highest LOD score on the X chromosome a prioritization of the two non-synonymous coding variants located on the X chromosome was made. Collectively, these data do not strongly support the non-coding BTA 7 variants as causative mutations.

**Table 1:** Variants detected by whole genome re-sequencing of an affected Pezzata Rossa cow.

Filtering step	Total number of variants^a^	Coding variants^b^
Variants in the whole genome	8,797,226	86,326
Variants in the critical intervals on BTA 7, 14, and X	361,134	1935
Variants in the critical intervals which were absent from 83 other cow genomes (local controls)	2593	21^c^
Variants in the critical intervals which were absent from 1119 genomes of the 1000 bull genomes project (global controls)	29^c^	2^d^

^a^ The sequences were compared to the reference genome (UMD3.1 assembly).

^b^ The following snpEFF categories of variants were considered as coding: SYNONYMOUS_CODING, NON_SYNONYMOUS_CODING, CODON_DELETION, CODON_INSERTION, CODON_CHANGE_PLUS_CODON_DELETION, CODON_CHANGE_PLUS_CODON_INSERTION, FRAME_SHIFT, EXON_DELETED, START_GAINED, START_LOST, STOP_GAINED, STOP_LOST, SPLICE_SITE_ACCEPTOR, SPLICE_SITE_DONOR.

^c^ These variants are listed in S1 Table.

^d^ Chr. X g.83,572,401 G>A (*ERCC6L)* and Chr X g.97,363,937 A>G (*TSR2*)

In addition to the SNP and short indel variant calling, large deletions contained in the candidate regions were searched for using 41 sequenced control cow genomes which were selected in order to have a genome-wide coverage of more than 10×. Of the 11,784 deletions detected across the whole genome of the sequenced cow, 49 were private structural variants occurring only in the genome of the affected cow. One heterozygous deletion found exclusively in the affected animal was detected situated in one of the mapped regions, at position 128,716,121 in chromosome X. The 4039 bp deletion is 2271 bp upstream of the first exon of the *membrane-bound transcription factor peptidase*, *site 2* (*MBTPS2*) gene. Subsequent PCR analysis confirmed the presence of this variant in case 1, in its sire and its three unaffected offspring, and its absence in the other family members including the other three affected cows.

The first of the two remaining private variants was a missense mutation in the bovine *ERCC6L* gene (c.54G>A) predicted to change an amino acid (p.A18T). The second private variant was a point mutation affecting the 5'-splice junction of exon 5 of the *TSR2* gene (c.441+226A>G). Both private variants were genotyped in all family members, and in two different cohorts of controls. The first cohort consisted of 1043 Pezzata Rossa cattle belonging to ten farms present in the same region including the farm of the four affected cows. All the Pezzata Rossa cattle were found to be free of streaked hairlessness. The second cohort consisted of 1682 animals of different cattle breeds from the DNA database present in our laboratory which had been collected during various studies. All four affected cows were heterozygous for both variants and the normal family members carried only the wild type allele. Both variants associated perfectly with the condition and were absent in all controls (Table 2).

**Table 2:** Association of the *TSR2* and *ERCC6L* variants with the streaked hairlessness phenotype.

	*TSR2* c.441+226A>G		*ERCC6L* c.54G>A	
	AA	AG	GG	AG
Affected cows		4		4
Normal family members	8		8	
Normal Pezzata Rossa controls	1043		1043	
Normal controls from other breeds	1682		1682	
Total	2733	4	2733	4

*In silico* analysis was then carried out on ERCC6L which predicted the p.A18T amino acid change as non-damaging with a Polyphen score of 0.002 out of 1. The predicted altered protein sequence of the mutant ERCC6L protein was aligned with the homologs of several other mammalian species which showed that the affected residue was not conserved across mammals (S5 Fig). Interestingly, threonine is present in the ERCC6L protein sequence of the African elephant. Furthermore, expression analysis in mice showed no specific pattern related to hair follicle development (S6 Fig). Collectively, these data do not support *ERCC6L* as the causative gene.

### A *TSR2* splice site mutation leads to aberrant transcripts in hairless skin

**Fig 4:**
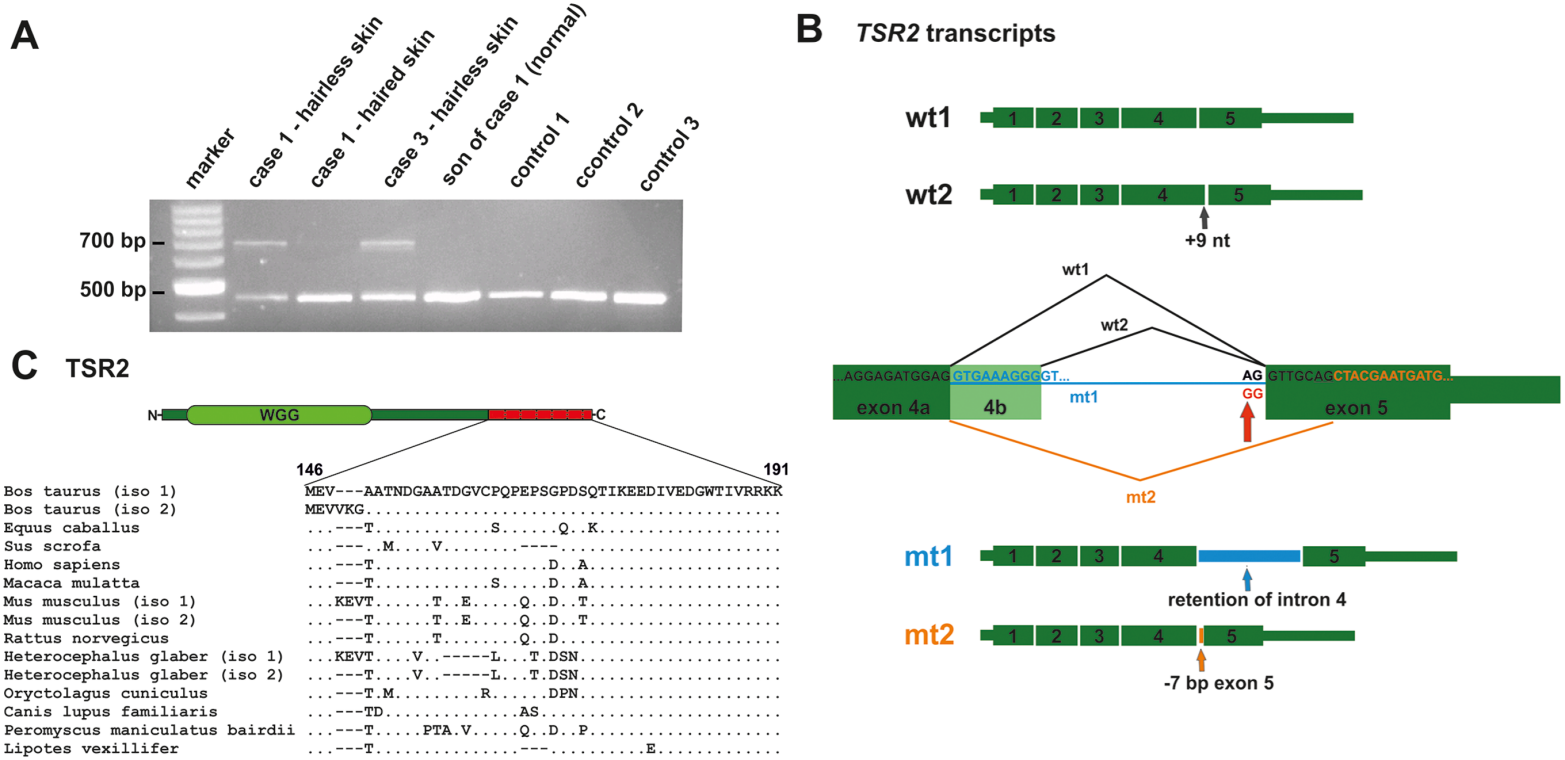
A *TSR2* splice site mutation leads to aberrant splicing in hairless skin. (A) An RT-PCR analysis of *TSR2* using primers located in exons 3 and 5 on the cDNA of the bovine skin of affected and unaffected animals. The lower band corresponds to the wild type transcript and was present in all the tissue samples examined. A second larger PCR product was present predominantly in the hairless skin of the affected cows. (B) Sequence analysis of the RT-PCR products revealed the presence of two wild type transcripts (*wt1* and *wt2*) and two mutant transcripts (*mt1* and *mt2*). The second wild type transcript includes 9 additional nucleotides of exon 4b. The 5'-splice junction mutation of exon 5 is indicated by the red arrow. Note that the splice acceptor site mutation results in two alterations: intron 4 retention and the alternative usage of a cryptic splice site in exon 5. (C) Bovine TSR2 protein. The conserved protein domain (WGG) is shown in light green and the predicted loss of the C-terminus is indicated in red. The terminal 19 amino acids are conserved in mammals, indicating a possible function role.

The *TSR2* mutation was predicted to affect splicing because it altered the conserved splice acceptor sequence AG at the 3’-end of intron 4, which was changed to GG. An RT-PCR was carried out to test the consequences of the 5'-splice junction mutation of exon 5. Therefore, primers located in exons 3 and 5 of *TSR2* were used to amplify cDNA from the affected and the unaffected skin of two cases, the normal skin of a related control (the first male offspring of case 1), and three unrelated controls (Fig 4A). The presence of two wild type transcripts was confirmed by Sanger sequencing in all tissues (Fig 4B). In the hairless skin of the affected cow, an additional prominent second band ~200 bp larger in size was detected. This additional band was also present in a much lower intensity in the normal haired skin of the affected cow. The RT-PCR products obtained from the hairless skin were cloned and Sanger sequencing of the various clones was performed. About 88% and ~2% of wild type transcript 1 and 2, respectively, and ~10% mutant transcripts were identified (Fig 4B and S7 Fig). The most common (~80%) mutant transcript 1 (*mt1*) was due to the retention of intron 4; splicing did not occur and exons 4 and 5 were separated by intron 4 in the transcript (c.441_442ins226). A less frequently occurring (~20%) second mutant transcript (*mt2*) was the result of alternate splicing, thereby activating a cryptic splice acceptor site 7 bp downstream which led to skipping the first 7 nucleotides of exon 5 (c.441_448del7) (Fig 4B and S7 Fig). Both mutant mRNAs contained a frameshift, and generated a premature stop codon predicted to truncate approximately 25% of the protein (*mt1*: p.Ala147Lysfs10*; *mt2*: p.Val146Leufs29*; S7 Fig).

### TSR2 is expressed in adult and fetal bovine skin and in developing and cycling murine hair follicles

**Fig 5:**
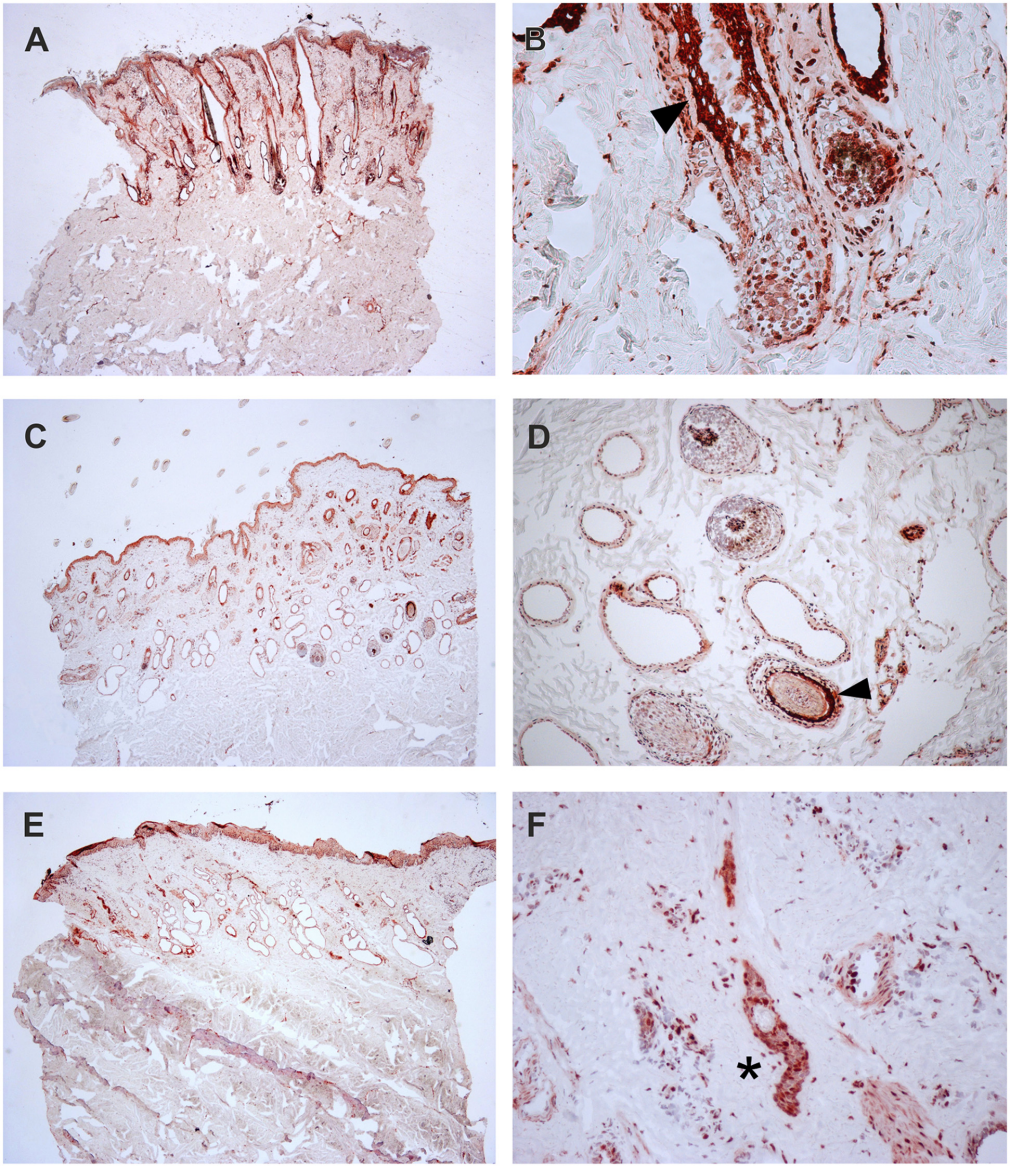
Expression of TSR2 protein in adult bovine skin. Immunohistochemistry carried out on skin biopsies using an anti-bovine TSR2 antibody. (A, B) Normally haired control cow (longitudinal section). A predominantly nuclear signal in the epidermal zone is present. Note that the bulge shows a particularly stronger signal (arrow). (C, D) Haired skin of an affected cow (transverse section). (E, F) Hairless skin of the same affected cow. The TSR2 expression in the haired skin corresponds to the expression in the normal cow; it is fainter in dysplastic hair follicles and absent in the inner root sheath of the affected skin (asterisk).

**Fig 6:**
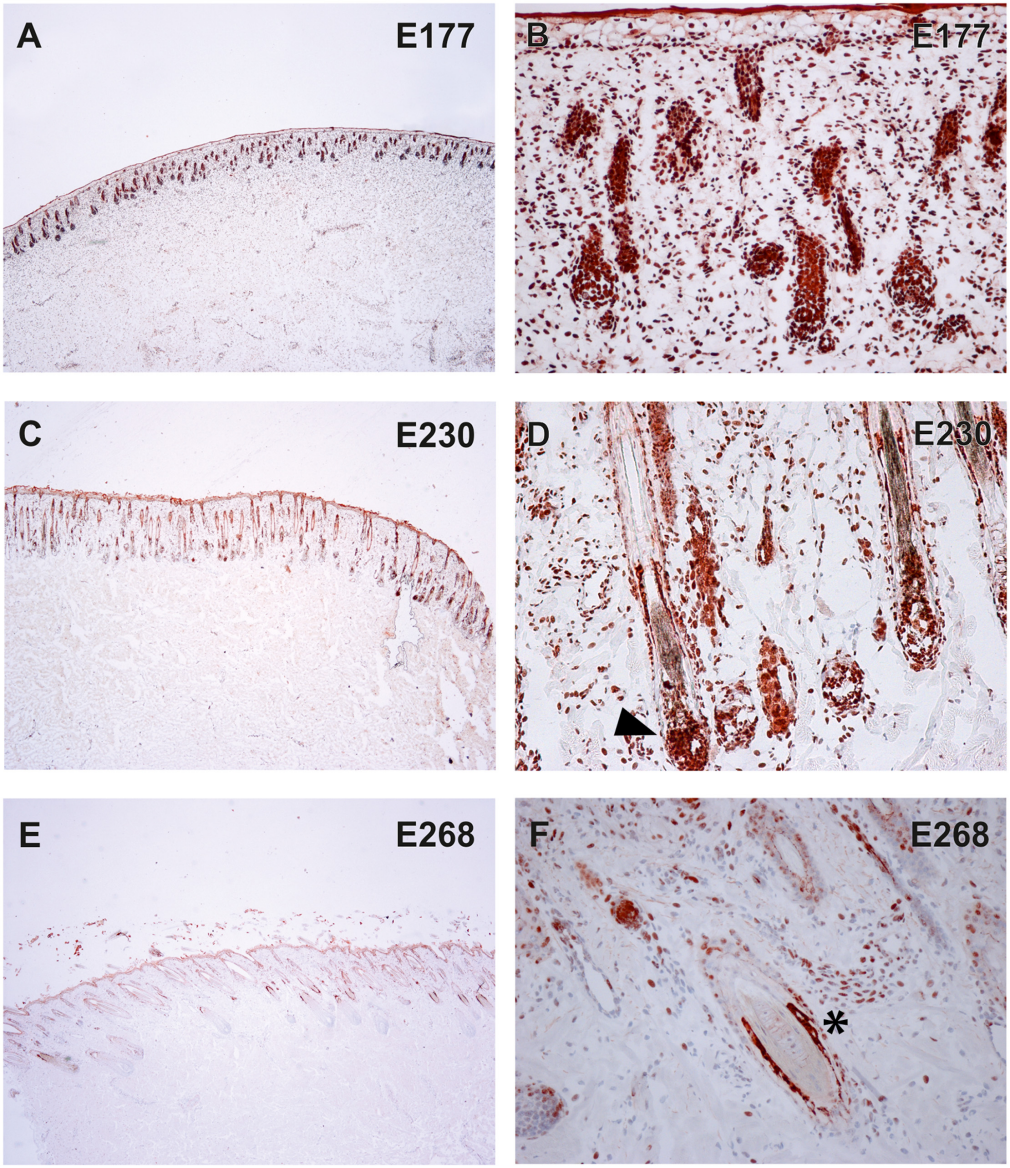
Expression of TSR2 protein in fetal bovine skin. Immunohistochemistry on wild-type fetal skin biopsies using an anti-bovine TSR2 antibody. The TSR2 protein is expressed in the epidermis and developing hair follicle. (A, B) Skin of a fetus at ~177 days of gestation (longitudinal section). (C, D) Skin of a fetus at ~230 days of gestation (longitudinal section). (E, F) Skin of a fetus at ~268 days of gestation (transverse section). Note the strong signal on the root sheath (day 230, arrow) and bulge (day 268, asterisk).

To verify the presence of the TSR2 protein in bovine skin, bovine fetal skin in different developmental stages, and hairless and normally haired skin of an affected and a control cow were used. Therefore, a species-specific antibody against the N-terminal part of the bovine protein was designed. A nuclear signal was detected in all epithelial cells in the hairless and the haired skin of the affected animal, and in the control cow (Fig 5). The TSR2 protein was strongly expressed in both cows within nuclei of epidermal and follicular keratinocytes, including cells of the hair bulbs as well as dermal papillae. In both cows, the protein expression was particularly strong in the root sheath. In the hairless skin areas of the affected cow, the root sheet was not present due to severe follicular atrophy. Nuclear expression was also observed in the majority of cell types present in the dermis including endothelial cells, epithelial cells of the sebaceous glands and the sweat glands, smooth muscle cells, infiltrating leukocytes and fibroblasts (Fig 5). The signal was not specific only for the hair follicle but also for the haired skin of both the case and the control which showed a denser signal on the upper part of the follicle toward the bulge (Fig 5). In addition, a nuclear TSR2 signal similar to that in adult cows was detected in all fetal samples (Fig 6). In the earlier fetal stage at day 177, TSR2 expression was detectable in all cells of the developing dermis. Interestingly, this signal was strongest in the hair bulb. At days 230 and 268, a strong signal appeared in the inner and outer root sheath of developing hair follicle (Fig 6).

**Fig 7:**
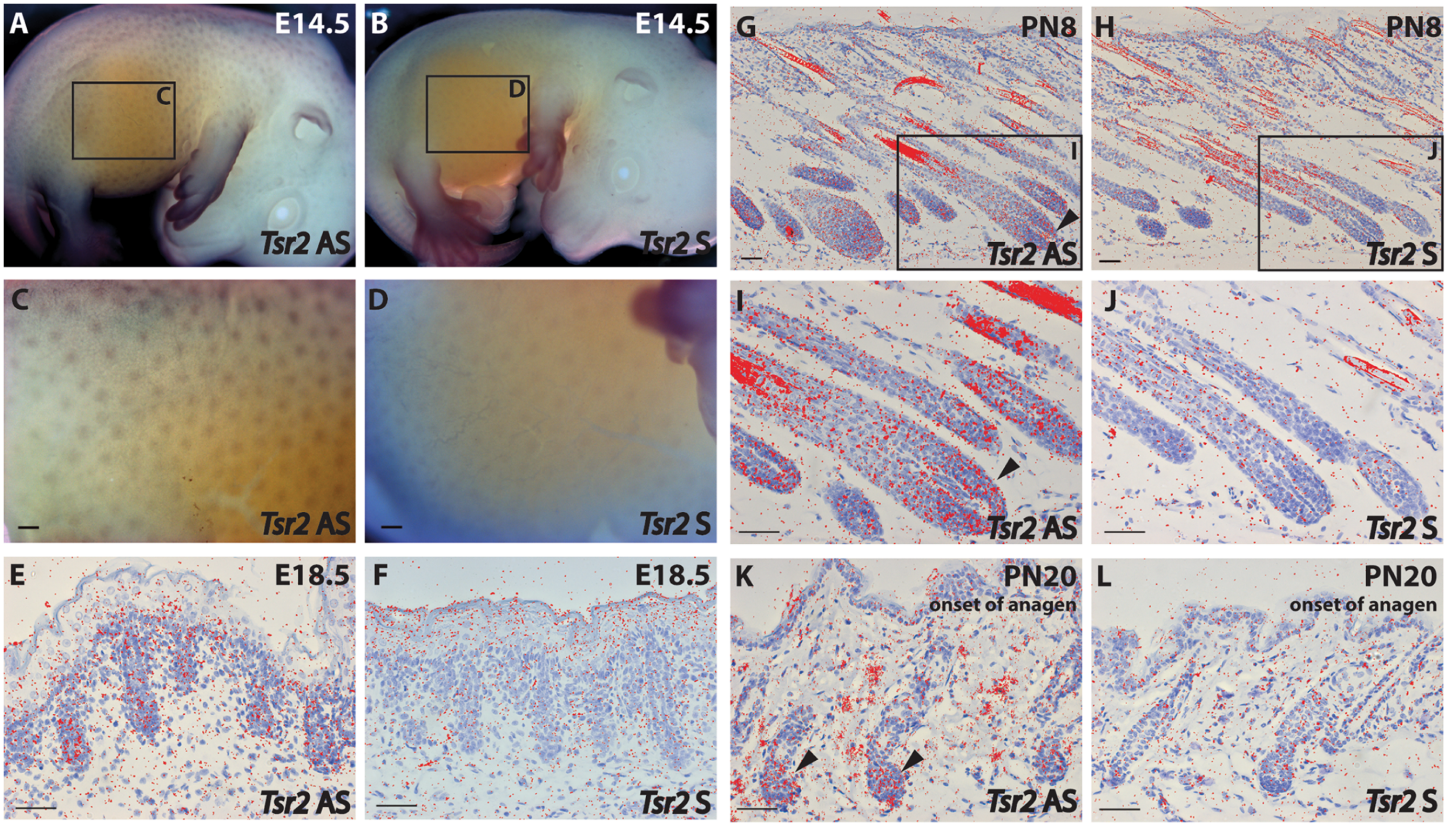
*Tsr2* is expressed in murine hair follicles. (A-D): Whole mount *in situ* hybridization of a mouse embryo at embryonic day (E) E14.5 with a digoxigenin-labeled *Tsr2* antisense (AS) (A, C) and sense (S) (B, D) probe. C and D are close-ups of the inserts shown in A and B, respectively. (E-L): *In situ* hybridization with a ^35^S-labeled *Tsr2* antisense probe (E, G, I, K) during embryonic (E18.5), and postnatal (PN) morphogenesis (PN8), and at the onset of anagen (PN20). A sense probe (F, J, H, L) was used as a control. I and H are close-ups of the inserts shown in G and J, respectively. Arrowheads mark the expression of *Tsr2* in the growing part of the hair follicle where proliferating cells reside. Scale bars are 200 (C, D) and 50 (E-L) μm.

To further elucidate TSR2 expression in the hair follicle, *in situ* hybridization (ISH) was performed at different stages of murine hair follicle morphogenesis and the postnatal hair cycle. *Tsr2* expression was detected in hair placodes using whole mount ISH at embryonic day 14.5, at the onset of hair development (Fig 7A and 7C) whereas the sense probe gave only a faint background signal (Fig 7B and 7D). *In situ* hybridization on sections with a ^35^S-labeled *Tsr2* probe also revealed low levels of expression in hair follicles during embryonic and postnatal growth phases as well as at the onset of anagen, the growth phase of the hair cycle (Fig 7E–7L). At all the stages analyzed *Tsr2* was enriched in the epithelial compartment of the hair follicle at sites where actively proliferating cells reside: in the growing edge of early postnatal hair follicles and in the pool of transit amplifying cells of the cycling hair follicles at the beginning of anagen. No signal was detected when ISH was performed with the *Tsr2* sense probe (Fig 7), confirming the specificity of the antisense probe.

## Discussion

### Streaked hairlessness in cattle—An example of skewed X-inactivation

A rare non-syndromic hairlessness phenotype was observed in cattle which could be explained by an X-linked mode of inheritance. This disorder occurred across three generations of a single family of Pezzata Rossa cattle and showed a striking similarity to a sex-linked inherited condition described as streaked hairlessness [11]. Eldridge and Atkinson reported affected females in a pedigree of Holstein Friesian cattle showing approximately perpendicular areas devoid of hair on various parts of the body with the hairless areas occurring in consistent patterns which were highly variable in size [11]. In comparison with the disease phenotype in our study, the only difference lay in the fact that the owner of the affected cows reported no differences in cold endurance which represented a difficult feature to assess due the difference among breeds and the zones in which the animals had been raised. The four related cows reported in the present study showed hairless streaks on various parts of the body regardless of the pigmentation. Interestingly, the affected streaks were S-shaped on the sides with a typical V shape near the center of the back occurring in a consistent pattern resembling the lines of Blaschko. Genetic mapping confirmed the initially suspected X-linkage and this congenital anomaly therefore added another example to the list of X-linked conditions with visible skin manifestations [6]. The characteristic appearance of the skin in the affected females is most probably correlated with the X-inactivation, as the lines of Blaschko are typically visible in heterozygous females of X-linked disorders affecting hair development such as incontinentia pigmenti, focal dermal hypoplasia or hypohidrotic ectodermal dysplasia [4–6]. The phenotype presented showed no typical features of ectodermal dysplasia since only hair and no other ectodermal derived organs, such as eccrine glands or teeth, were affected. Notably, the anomaly was restricted to the regional absence of only hair follicles and sebaceous glands.

The manifestation of X-linked phenotypes depends largely on the way in which cells subsequently divide and migrate, and is best studied in skin diseases [6]. The archetypal cutaneous pattern described by the dermatologist Alfred Blaschko [1] was later explained by the mosaicism which resulted from XCI in migrated ectodermal skin cells of females [4]. It was hypothesized that the four affected females who were heterozygous for private mutations on the X-chromosome showed varying phenotype expression affecting only small parts of their skin due to skewed X-chromosome inactivation (XCI). This is known to influence the appearance and severity of X-linked traits in heterozygous females by selective skewing in favor of cells which express the wild type alleles [6, 7]. In the earlier report of bovine streaked hairlessness lethality in males carrying a copy of the putative X-linked mutation was assumed, thus supporting our hypothesis that the affected gene was subject to XCI.

### A candidate causative *TSR2* splice site mutation

Evidence that the X-linked streaked hairlessness phenotype is likely caused by a disruptive mutation disturbing the normal splicing of the *TSR2* gene was provided. During our study, positional cloning using linkage analysis and mutation analysis using whole genome sequencing were combined. Access to sequenced genomes of other cattle breeds and to the 1000 bull variant database [13] was very useful in detecting the disease-associated mutations. These filter steps allowed us to significantly reduce the number of associated variants within the critical regions. The investigation was not restricted to SNPs or short indels affecting annotated genes since the observed phenotype was mild and unclear in its definition. It was therefore taken into account that other types of mutations, such as larger structural variants, could cause the disorder. The 2.3 kb deletion identified upstream of the *MBTPS2* gene, a candidate gene for a skin condition [14], was finally excluded as potentially causative due to its presence in non-affected family members. Nonetheless, two private, perfectly associated single nucleotide sequence variants remained which were located in two X chromosomal genes: *ERCC6L* and *TSR2*. These two variants were subsequently genotyped in more than a thousand animals of the affected Pezzata Rossa breed but they remained private for the four affected cows and obviously occurred in complete linkage disequilibrium although they were located nearly 13 Mb apart. The *ERCC6L* gene encoded a DNA helicase which acted as an essential component of the spindle assembly checkpoint. The amino acid substitution occurred in a residue located in a non-conserved region, and the mutant residue was found in the wild type protein sequence of the African elephant. Furthermore, the experiments in developing mice showed no specific expression in hair follicles. For this reason, it was concluded that the missense mutation in *ERCC6L* was unlikely to be causative for the condition observed. The remaining mutation in the *TSR2* splicing site was shown to lead to two mutant transcripts predominantly expressed in the hairless skin of the affected cows. Neither mutant transcript contained the terminal part of the TSR2 protein the function of which is unknown. To date, the only known putative functional WGG domain is situated in the N-terminal region of the protein. It was therefore concluded that this *TSR2* variant present on the X chromosome represented a candidate causal mutation for the naturally occurring condition.

### The rRNA accumulating TSR2 protein is implied to play a significant role during hair follicle development

The exact function of the *TSR2* gene during hair follicle development had not been clarified until now. In order to validate whether *TSR2* might be a reasonable functional candidate gene for the observed disorder, its expression in different stages of bovine and murine hair follicle morphogenesis and cycles was analyzed, including the time periods during which the ectodermal differentiation leading to the formation of hair takes place.

Using tissue from the affected and control cows, TSR2 protein expression was detected in adult bovine skin. A clear signal was detected in the hair follicle, confirming the presence of the associated protein in the tissue affected by the condition. In order to investigate the presence of the protein during development, samples from bovine fetal skin from a previous study estimated as being from day 177, 230 and 268 of gestation were used [15]. These three time points were chosen because they represented critical moments during hair follicle development: in the developing bovine embryo, one can detect a formed papilla between days 140–180, emerged hair between days 220–260 and the end of follicle length growth between days 240–280 [16]. A hair follicle is a dynamic self-renewing organ which periodically regenerates through cycles of regression (catagen), rest (telogen) and new growth (anagen). Hair follicle development is initiated during embryogenesis by the formation of an epithelial thickening (a placode) and an associated mesenchymal condensate (a dermal papilla). After the initial period, the hair follicle grows downwards into the mesenchyme and, once morphogenesis is completed, it enters the first hair cycle [17]. In mice, morphogenesis is completed by postnatal (PN) days 13–15, first catagen is initiated at ~PN17, and first anagen at ~PN20 [17, 18]. The fact that *Tsr2* mRNA was expressed in mouse hair follicles at the initial stage of development, in the growing hair follicles during embryogenesis and in anagen follicles in the adult skin at the site where proliferating progenitor cells reside was shown. Defects in cell proliferation during anagen could lead to impaired hair follicle down growth. Expression at the site of the proliferating cells in developing murine hair follicles suggests that *Tsr2* could be important for hair growth.

Currently, little is known regarding the cellular function of TSR2. Studies involving yeast have suggested a role in 20S rRNA processing [19, 20]. Cytoplasmic cleavage of the 20S pre-rRNA to 18S is critical for the maturation of 40S subunits; the depletion of *Tsr1*, the paralog which is essential to ribosome biogenesis [21, 22], and *Tsr2* all lead to 20S accumulation [19, 23–27]. Fassio et al. found TSR2 nonessential for yeast survival, but deletion resulted in slow growth with a doubling time of ∼2.5 hrs in addition to a prominent 20S accumulation and a corresponding 18S deficit [20]. The paralog TSR1 is detected in yeast in both the nucleus and the cytoplasm, but is predominantly nuclear in exponentially growing cells [22–27]. A recent paper of Schütz et al [28] reported better insights into the function of the protein in yeast; it was shown that TSR2 bound released protein eS26, shielded it from proteolysis, and ensured its safe delivery to the 90S pre-ribosome. The authors defined the role of TSR2 protein as a nuclear carrier; its role is hypothesized to securely connect the nuclear import machinery with pathways which deposit r-proteins onto developing pre-ribosomal particles. A mutation within eS26 has been associated with Klippel-Feil syndrome in Diamond-Blackfan anemia [29–31]. A *TSR2* missense mutation affecting the highly conserved predicted WGG domain (of unknown function) was reported to be associated with Diamond-Blackfan anemia with mandibulofacial dysostosis (Treacher-Collins syndrome)–a congenital anomaly involving absent external auditory canals and abnormal middle ears, micrognathia, unilateral cryptorchidism and a submucous cleft palate but no known hair phenotype [32]. Of note, the candidate mutation identified as causing streaked hairlessness in cattle did not affect the WGG domain. However it resulted in the formation of a C-terminal truncated version of the TSR2 protein. Notably, the C-terminal part of TSR2 is highly conserved among mammals, thereby suggesting a potential functional role of this domain, although no role has been inferred until now. We therefore speculate that the C-terminal part had a previously unknown important function during hair follicle development. In addition to its role in rRNA biogenesis, TSR2 is reportedly associated with other cellular processes. Behrends et al. identified TSR2 as one of the candidate interactors in the human autophagy system [33] whereas He et al. [34] reported that overexpression of TSR2 in human epidermal HEp-2 cells inhibited the transcriptional activity of NF-kappaB and induced HEp-2 cell apoptosis. The effect of the mutation appears to be circumscribed to the skin, even if *TSR2* is supposed to be expressed ubiquitously. Cell or tissue specificity of the phenotype caused by a mutation in a gene expressed in the entire organism is not unknown, especially if some sort of compensation mechanism is not specifically available in the affected tissue [35]. In addition, probably skewed X-inactivation in favor of the cells expressing the wild type allele played an important role in the development and severity of the phenotype. The outcome of the study provided the first insights of the possible involvement of the TSR2 protein with a tissue or in a cell-specific manner. The TSR2 protein is potentially involved in several cell pathways, and the dynamics behind its relevance in several cell processes has yet to be unraveled.

## Materials and Methods

### Ethics statement

All animal research was conducted according to national and international guidelines for animal welfare. No permit number was necessary for the cattle as this study used naturally occurring cases. The bovine samples used were taken from different cattle farms in Italy, and all cattle owners agreed that the samples could be used in the study. The collection of fetal tissue, already used in previous studies [15], was carried out at a local government-authorized slaughterhouse in Switzerland since only a small number of pregnant cows are routinely slaughtered. All experiments involving mice were carried out in accordance with the guidelines and approval of the National Animal Experiment Board of Finland, the institute issuing the license is the Laboratory Animal Center of the University of Helsinki, and the license number is KEK13-020.

### Animals and sample gathering

Blood samples were collected from four affected Pezzata Rossa cows from the same farm. Genotyping of these cases was carried out using BovineHD BeadChip (illumina), including 777,962 evenly distributed SNPs at Geneseek (S1 Dataset). In addition, blood and semen samples were collected from eight cattle recorded as mates, parents and offspring of the affected cows (Fig 2A). A total of 1043 blood samples were collected from Pezzata Rossa cows from ten different farms in the region. The stored DNA samples from 1682 cattle belonging to several breeds previously subject of study, mainly Chianina, Romagnola, Simmental and Holstein Friesian were used. During the mutation analysis, 83 genomes of normal cattle from 17 genetically diverse *Bos taurus* breeds were used as local control cohort. The recent sequence variant database containing 1119 already sequenced genomes of the ongoing 1000 bull genomes project [13] was used as global control cohort during filtering for private variants of the sequenced affected cow.

### Histopathological examination

Eight millimeter skin punch biopsies were obtained from two affected cows and one normal offspring after subcutaneous injection of 2% lidocaine. The samples were collected at different sites, both at the level of the hairless streaks (lesional skin) and from grossly normal haired skin (unaffected skin), and from the border between haired and non-haired skin. All specimens were fixed in 4% buffered formaldehyde solution for histopathological examination or frozen at -80°C. After processing, they were embedded in paraffin, sectioned at 4 μm and stained with haematoxylin and eosin.

### Linkage analysis

PLINK v. 1.07 software [36] was used to prepare the dataset for the linkage analysis using the—cow command to take into account the species specific number of chromosomes. The genotype data was pruned for the subsequently performed linkage analysis: (1) to remove SNPs with more than 10% missing genotype calls (—geno 0.1); (2) to exclude uninformative SNPs with a minor allele frequency below 5% (—maf 0.05); and (3) to exclude SNPs which exceeds the Hardy-Weinberg disequilibrium p-value of 0.0001 (—hwe 0.0001). MERLIN v 1.1.2 software [37] was used to analyze the dataset and carry out the linkage analysis. The—error was carried out in order to obtain a list of Mendelian errors and, hence, the SNPs to be excluded from the dataset. For all the autosomes, the multipoint LOD scores were calculated in a monoallelic autosomal dominant trait model, assuming complete penetrance. For the calculations, a frequency of 0.15 for the mutated allele was assumed. In addition, the same parameters were used to analyze the X chromosome using MINX (part of the MERLIN package) which implements X-chromosome-specific versions of the functions provided by standard MERLIN. Due to the missing parents and the small number of cases, any result showing a positive LOD score was hypothesized to be suggestive of linkage. Graphs were traced with the—pdf command. Haplotypes were estimated using MERLIN by means of the—best command chromosome-by-chromosome (after extraction of each single chromosome from the dataset with PLINK using the—chr command). Haplotypes and markers were visualized using Haplopainter [38].

### Cytogenetics

Heparinized blood samples were collected from one normal male calf and three affected females of the Pezzata Rossa cattle breed. The lymphocytes were cultured in 5 ml of RPMI-1640 medium containing 15% FCS, 1% L-glutamine (200 mM), 0.6% heparin (50 mg/ml), 0.8% pokeweed mitogen (80 μg/ml), 0.1% penicillin (20.000 U/ml) and 0.1% streptomycin (20 mg/ml) for 72 h at 37°C. Two hundred microliters of Colcemide (10 μg/ml) were added to the cultures for the last 45 min of culturing. Incubation in a hypotonic solution of KCl (75 mM) at 37°C was carried out for 20 minutes, and the chromosomes were then fixed three times in a methanol:acetic acid solution (3:1) and stored at -20°C. For each animal, 100 Giemsa-stained metaphases were analyzed using a Zeiss Axio Imager Z1 microscope. For each animal, ten metaphases were captured, and karyograms were prepared using IKAROS software (Metasystems).

### Whole genome re-sequencing

A fragment library with a 300 bp insert size was prepared and one lane of illumina HiSeq2000 paired-end reads (2x 100 bp) was collected; the fastq files were created using Casava 1.8. A total of 767,575,378 100 bp paired-end reads were collected from a shotgun fragment library corresponding to roughly 28× coverage of the genome. The paired-end reads were then mapped to the cow reference genome UMD3.1/bosTau6 and aligned using Burrows-Wheeler Aligner (BWA) version 0.5.9-r16 [39] with default settings. The mapping showed that 756,619,120 reads (98.6%) had unique mapping positions. The SAM file generated by BWA was then converted to BAM and the reads were sorted by chromosome using samtools [40]. The PCR duplicates were marked using Picard tools (http://sourceforge.net/projects/picard/). The Genome Analysis Tool Kit (GATK version 2.4.9, [41]) was used to carry out local realignment and to produce a cleaned BAM file. Variant calls were then made with the unified genotyper module of GATK. The variant data for each sample was obtained in variant call format (version 4.0) as were raw calls for all samples and sites flagged using the variant filtration module of GATK. Variant filtration was carried out, following the best practice documentation of GATK version 4. The snpEFF software [42], together with the UMD3.1/bosTau Ensembl annotation, was used to predict the functional effects of the variants detected. The genome data was made freely available under accession no. PRJEB8226 at the European Nucleotide Archive [43]. The Delly package [44] was used to detect structural variants in the cleaned BAM files. In order to avoid missing large inserts, deletions and false positives, all the variants detected were also manually inspected in the candidate region using 41 control genomes.

### Genotyping of variants

The associated variants were genotyped by the re-sequencing of targeted PCR products using Sanger sequencing technology. The primers were designed using PRIMER3 [45]. The PCR products were amplified with AmpliTaqGold360Mastermix (Life Technologies), and the products were directly sequenced using the PCR primers on an ABI 3730 capillary sequencer (Life Technologies) after treatment with exonuclease I (NEB) and rapid alkaline phosphatase (Roche). The sequence data were analyzed using Sequencher 5.1 (GeneCodes).

### Protein sequence analysis

Sequence alignment and mutation impact calculation for the ERCC6L mutant protein mutation was carried out with the prediction tool Polyphen 2 [46]. Sequence alignment was carried out using ClustalW [47].

### RNA extraction and RT-PCR

The RNA was extracted from skin tissues using the RNeasy mini kit (Qiagen). The tissue was first finely crushed in TRIZOL (Ambion) using mechanical means, chloroform was then added and the RNA was separated by means of centrifugation. Additional passages were carried out as described by the manufacturer. The RNA was cleared of genomic DNA contamination using the Quantitect Reverse Transcription Kit (Qiagen). The same kit was used to synthetize cDNA, as described by the manufacturer. An RT-PCR was carried out using AmpliTaqGold360Mastermix (Life Technologies). The RT-PCR products were sequenced as described above. The products were ligated to TOPO TA cloning plasmids pCRII (Invitrogen), as described by the manufacturer.

### *In situ* hybridization

For whole mount ISH, E14.5 mouse embryos were dissected, fixed in 4% paraformaldehyde PFA, and dehydrated using methanol series. Whole-mount *in situ* hybridization with a digoxygenin-labelled *Tsr2* probe was performed according to a standard protocol using InsituProVS instrument (Intavis Bioanalytical Instruments) [48]. The *Tsr2* antisense and sense probes corresponded to nucleotides 40–822 of NM_175146.4. The probes were detected with BM Purple AP Substrate Precipitating Solution (Roche Applied Science). For radioactive ISH, mouse back skins were fixed overnight in 4% PFA, dehydrated in ethanol, embedded in paraffin and sectioned at 5 μm. Radioactive *in situ* hybridization with a ^35^S-UTP (Amersham)-labeled *Tsr2* probe was carried out according to standard protocol [48].

### Western blotting

To generate the expression plasmids encoding the wild type and mutant (mt1) proteins (pCI-W, pCI-M), the two relevant sequences were synthesized (Eurofins). The plasmids were HA-tagged (peptide YPYDVPDYA). Next, pCI-RFP-HA-W and pCI-RFP-HA-M, and the plasmids were generated by the PCR amplification vector and were subsequently cloned into the pCI-RFP-Linker-HA-cleaved plasmid. Competent XL10-Gold Ultracompetent Cells were transformed as described above, and the plasmid was recovered and used to transfect the Vero cells. Vero cells expressing the two constructs were grown in Dulbecco’s modified Eagle’s medium (Invitrogen) with 10% fetal calf serum at 37°C in the presence of 5% CO_2_.

A positive and specific signal was obtained for the proteins translated from both transcripts from the wild-type and mutant vectors expressed in vero cells. The antibodies were designed against the N terminal part of the TSR2 protein and synthesized in rabbits (ProteoGenix). Western blots were carried out as previously described [49]. Transfected cells were washed twice with cold PBS before adding 150 μL of lysis buffer [10 mM Tris, pH 7.4, 150 mM NaCl, 1% deoxycholate, 1% Triton X-100, 0.1% sodium dodecyl sulfate (SDS)] with a complete protease inhibitor (Roche). After incubation for 20 min at 4°C, the lysates were cleared by centrifugation at 5000 g for 15 min at 4°C, and the supernatant was mixed with an equal amount of Laemmli sample buffer (Bio-Rad) containing 100 mM dithiothreitol, subsequently boiled at 95°C for 5 min, and fractionated on 8% or 10% SDS-polyacrylamide gel under denaturing conditions. The separated proteins were transferred to nitrocellulose membranes by electroblotting. The membranes were then incubated with polyclonal rabbit anti-CDV antisera. Following incubation with a peroxydase-conjugated secondary antibody, the membranes were treated with an enhanced chemiluminescence (ECL) kit (Amersham) according to the manufacturer’s instructions.

### Immunofluorescence

The transfected cells were washed with PBS and fixed with 500 μl 4% PFA (paraformaldehyde) for 20 min at room temperature. After a PBS wash, the cells were permeabilized with 500 μl to 1 ml of 2% Triton in PBS for 20 min at room temperature. After a PBS wash, they were incubated for one hour with primary antibody 1 μg/ml per well. After a PBS wash, secondary antibody goat anti-rabbit antibody Alexa fluor was diluted 1/1000 and incubated one hour before acquisition.

### Immunohistochemistry

Skin samples from normal fetuses collected at the slaughterhouse, and from affected (haired and hairless areas) and unaffected cows were embedded in Optimal Cutting Temperature compound (OCT) and were snap frozen by immersion in 2-methylbutane (59080; Sigma—Aldrich), which was chilled in liquid nitrogen. The frozen tissue blocks were stored at -80°C until cutting. Immunohistochemistry was carried out as previously described [50]. Briefly, cryostat sections were fixed in ice-cold acetone for 3 min and endogenous peroxidase activity was quenched by incubation with 3% hydrogen peroxide in methanol for 30 min. A protein block was obtained by applying 10% normal goat serum in PBS for 30 min. The slides were incubated with the primary antibody (the in-house produced polyclonal rabbit antibody) at 4◦C overnight. The positive reactions were detected with a LSAB-kit (Dako) according to the manufacturer’s instructions.

## Supporting Information

S1 Dataset SNP genotypes of the cattle family. Genotypes of 12 family members at 777,962 SNPs of the BovineHD BeadChip (illumina) are presented in the transposed file format (.tfam and.tped) of PLINK software [36].
(ZIP)

S1 Fig Phenotype differences of streaked hairlessness in Pezzata Rossa cattle. (A) Right side of case 1. (B, C, D) Hairless regions in cases 2, 3 and 4. Note the differently expressed phenotype between the affected cows. In case 4 (D), the V shaped pattern and the S-shaped pattern on the sides are particularly evident.
(TIF)

S2 Fig Histological findings of skin samples from a cow with streaked hairlessness under higher magnification. (A) Border between hairless and haired skin. Note that several hair follicles and sebaceous glands are present, and that the follicles are dysplastic. The dysplasia is characterized by distorted follicles and hair fragments within the follicular lumen. (B) Note that only one infundibulum is present in the hairless skin of the same cow and that the sebaceous glands are missing. Haematoxylin and eosin staining, magnification 200X.
(TIF)

S3 Fig Cytogenetic analysis. Metaphase spreads (left) and karyotypes (right) of three affected cows (A, B, C) and a normal male offspring of case 1 (D).
(TIF)

S4 Fig Parametric linkage analysis assuming monogenic dominant inheritance. Note the positive results on chromosomes 7 (maximum LOD score of 0.203), 14 (maximum LOD score of 1.203) and X (maximum LOD score of 1.405). Alpha score (alpha being the 'a priori' proportion of linked pedigrees) for LOD scores greater than 0 is 1.
(TIF)

S5 Fig Multi-species alignment of the N-terminal ERCC6L protein sequence. Note the lack of conservation of the affected residue (p.A18T) across mammalia.
(TIF)

S6 Fig Lack of *Ercc6l* expression in the developing mouse hair follicle. (A-F): Whole mount in situ hybridization of mouse embryos with dig-labelled *Erccl6l* antisense (AS) (A, C) and sense (S) (B, D) probes. No *Ercc6l*-specific signal was detected in the hair placodes at E14.5. C and D are close-ups of the inserts shown in A and B, respectively. (G-H): *In situ* hybridization with ^35^S-labeled *Ercc6l* AS (E, G) and S (F, H) probes during embryonic (E18.5) and postnatal (PN8) hair follicle morphogenesis. No specific signal was detected with more sensitive radioactive ISH technique, indicating the absence of *Ercc6l* transcripts in hair follicles at the stages analyzed. Scale bars are 200 μm.
(TIF)

S7 Fig Bovine *TSR2* transcripts and TSR2 protein sequences. (A) Open reading frame (ORF) of both experimentally-detected wild type *TSR2* transcripts and their encoded protein sequence. Sequences corresponding to exon 5 are shown in green. Note the underlined 9 nucleotides corresponding to exon 4b encoding three additional residues of TSR2 isoform 2. (B) Coding sequence and deduced protein sequence of two experimentally detected mutant *TSR2* transcripts. The retained intron 4 sequence is shown in blue until a premature stop codon is reached. The utilization of an alternate AG splice site immediately downstream from the normal splice site, which is mutated, leads to a 7 nt shorter exon 5 (indicated by dots). The resultant mutant transcript 2 contains a frameshift which is predictive of a truncation.
(PDF)

S1 Table List of filtered sequence variants. (PDF)
