# Correction: Hairless Streaks in Cattle Implicate TSR2 in Early Hair Follicle Formation

**DOI:** 10.1371/journal.pgen.1005688

**Published:** 2016-05-02

**Authors:** Leonardo Murgiano, Vera Shirokova, Monika Maria Welle, Vidhya Jagannathan, Philippe Plattet, Anna Oevermann, Aldona Pienkowska-Schelling, Daniele Gallo, Arcangelo Gentile, Marja L. Mikkola, Cord Drögemüller

The tenth author's name is spelled incorrectly. The correct name is: Marja L. Mikkola.
