# Supplementary figures and images for: Hairless Streaks in Cattle Implicate TSR2 in Early Hair Follicle Formation
Source: PLoS Genet. 2015 Jul 23;11(7):e1005427. doi: 10.1371/journal.pgen.1005427 (PMC4512707; doi:10.1371/journal.pgen.1005427)

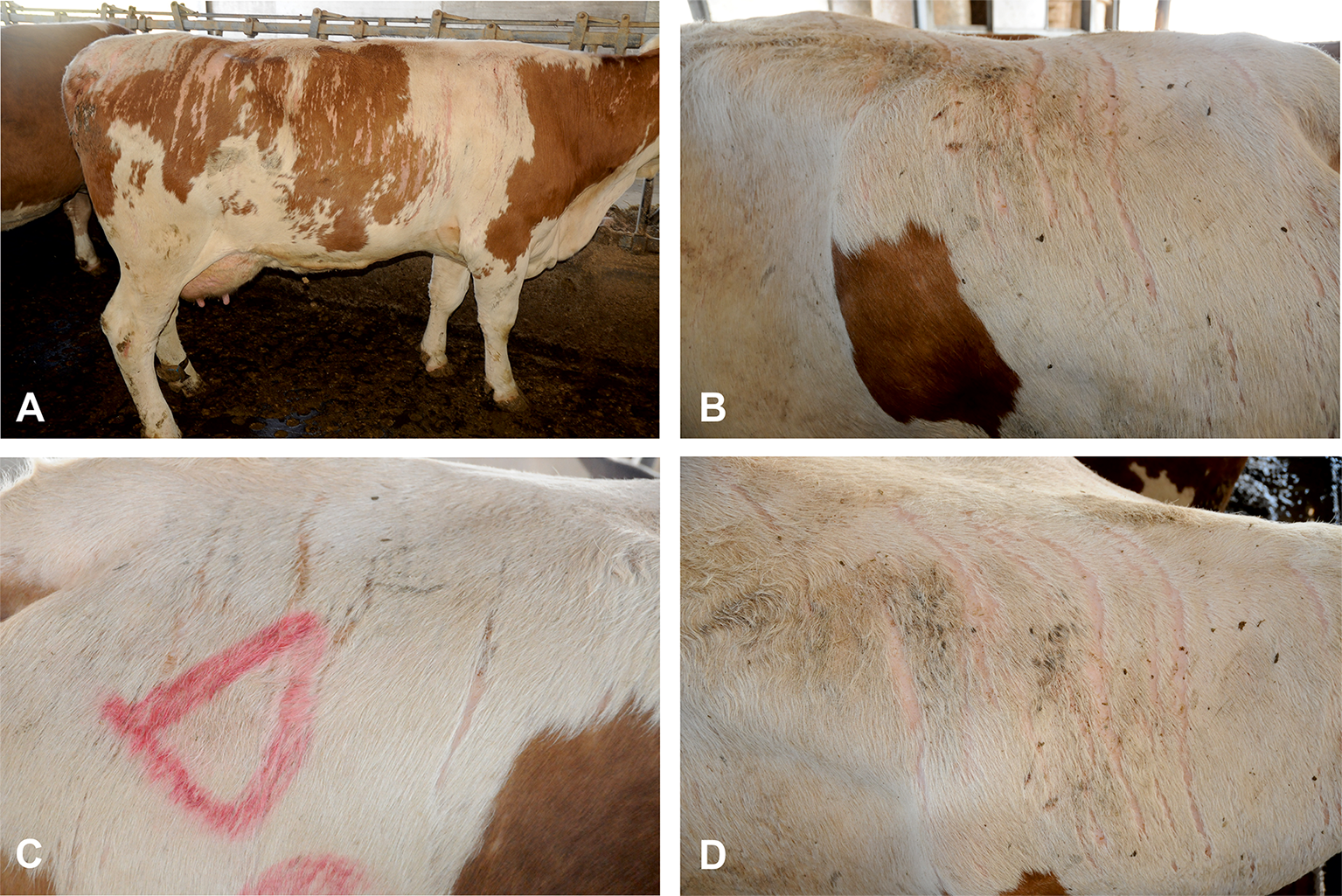

Supplement: S1 Fig — (A) Right side of case 1. (B, C, D) Hairless regions in cases 2, 3 and 4. Note the differently expressed phenotype between the affected cows. In case 4 (D), the V shaped pattern and the S-shaped pattern on the sides are particularly evident. (TIF) [file pgen.1005427.s002.tif]

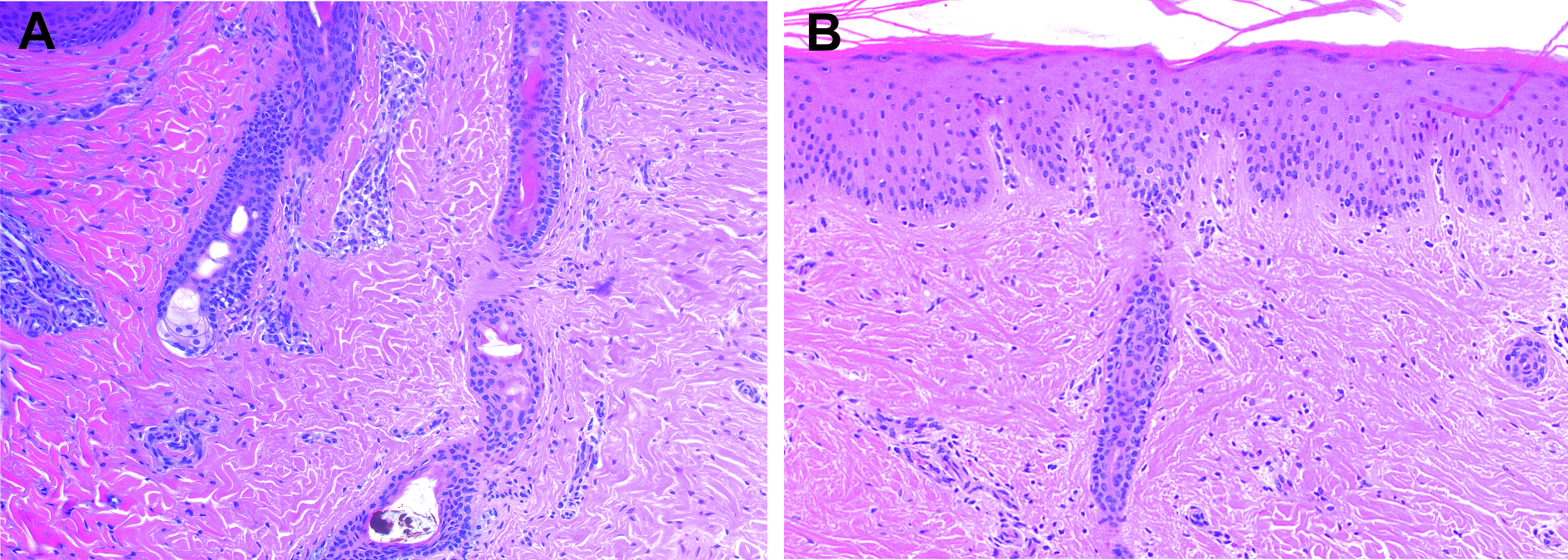

Supplement: S2 Fig — (A) Border between hairless and haired skin. Note that several hair follicles and sebaceous glands are present, and that the follicles are dysplastic. The dysplasia is characterized by distorted follicles and hair fragments within the follicular lumen. (B) Note that only one infundibulum is present in the hairless skin of the same cow and that the sebaceous glands are missing. Haematoxylin and eosin staining, magnification 200X. (TIF) [file pgen.1005427.s003.tif]

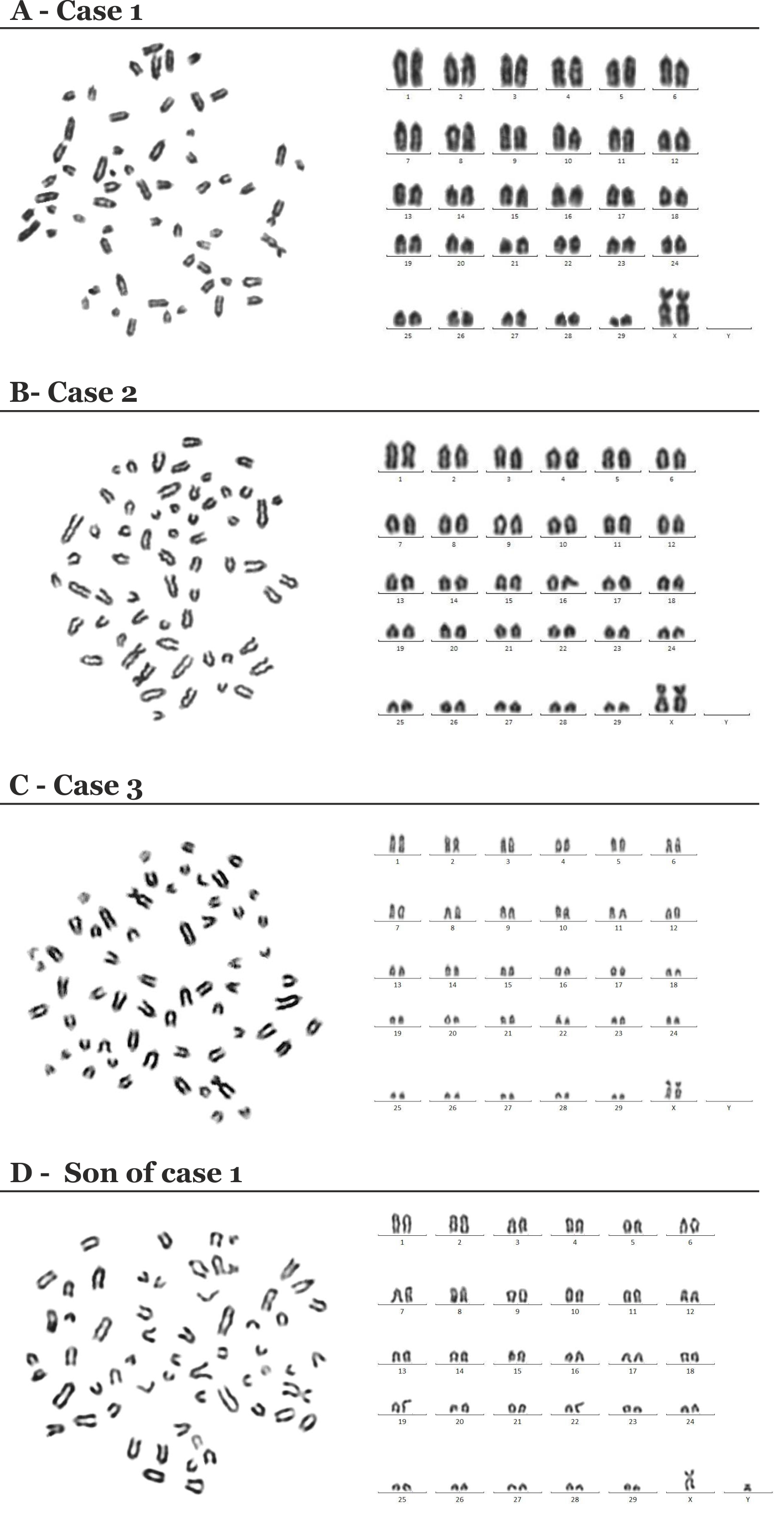

Supplement: S3 Fig — Metaphase spreads (left) and karyotypes (right) of three affected cows (A, B, C) and a normal male offspring of case 1 (D). (TIF) [file pgen.1005427.s004.tif]

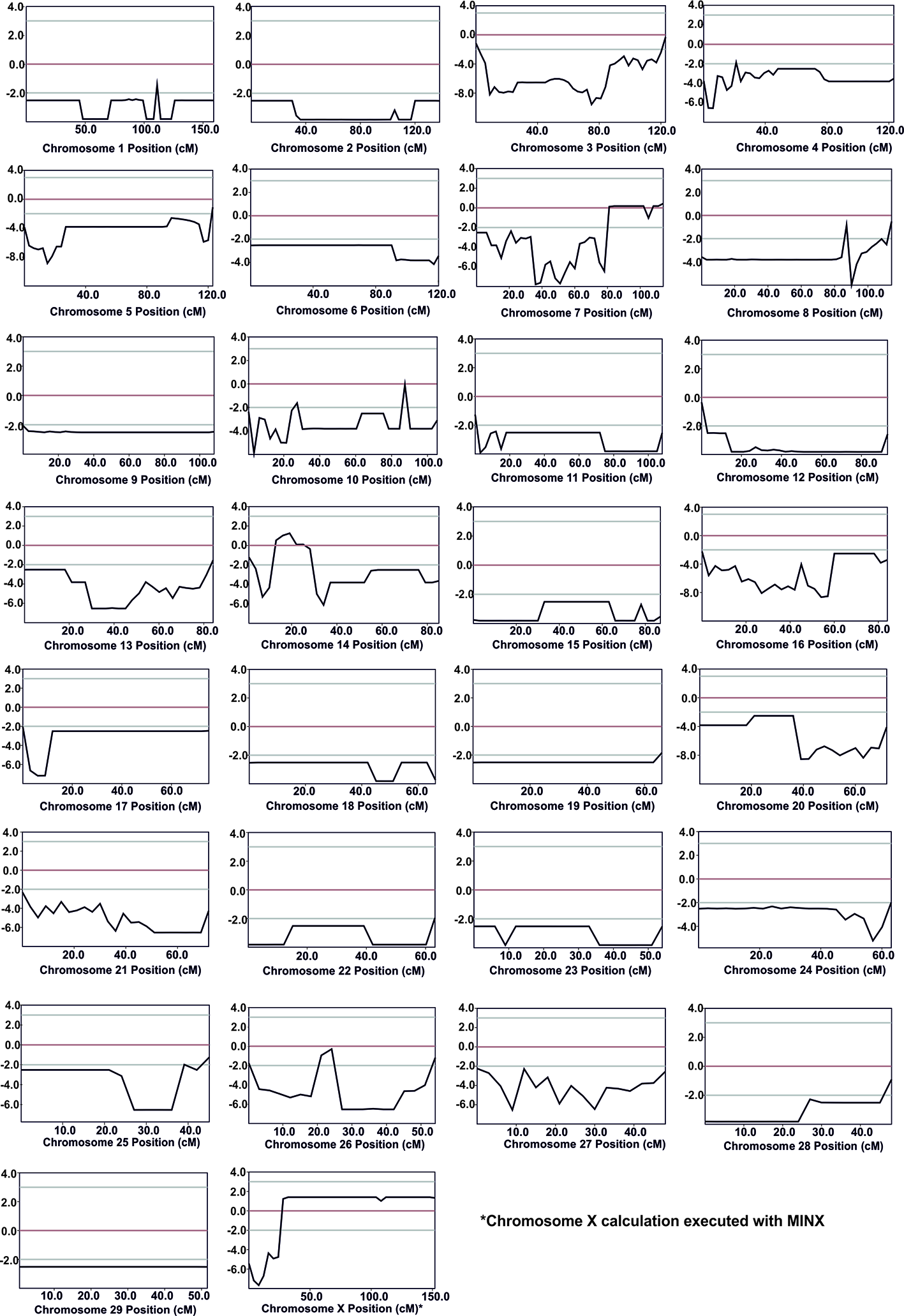

Supplement: S4 Fig — Note the positive results on chromosomes 7 (maximum LOD score of 0.203), 14 (maximum LOD score of 1.203) and X (maximum LOD score of 1.405). Alpha score (alpha being the 'a priori' proportion of linked pedigrees) for LOD scores greater than 0 is 1. (TIF) [file pgen.1005427.s005.tif]

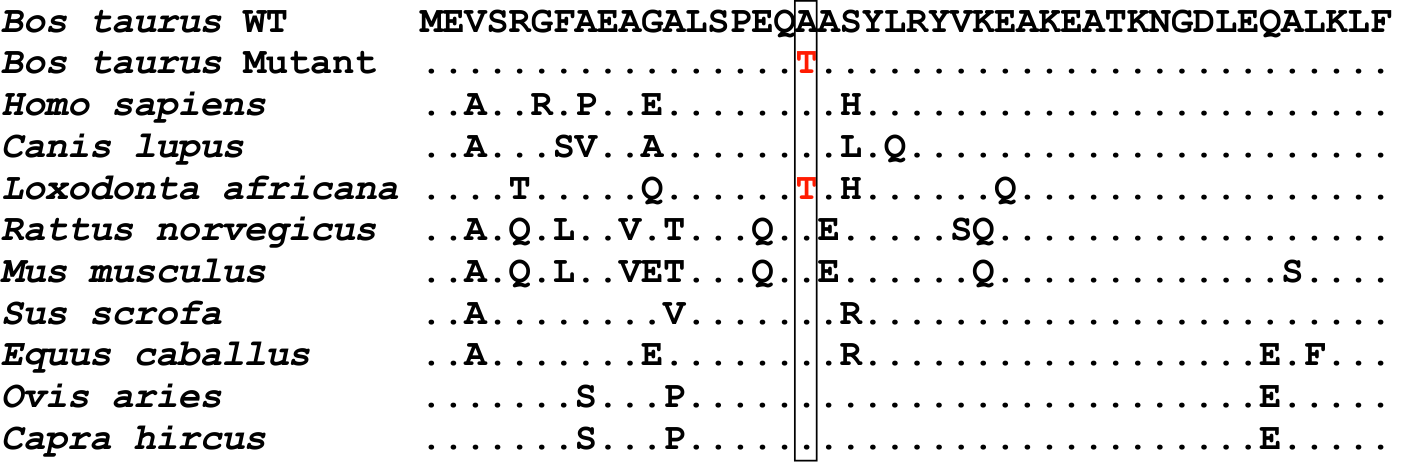

Supplement: S5 Fig — Note the lack of conservation of the affected residue (p.A18T) across mammalia. (TIF) [file pgen.1005427.s006.tif]

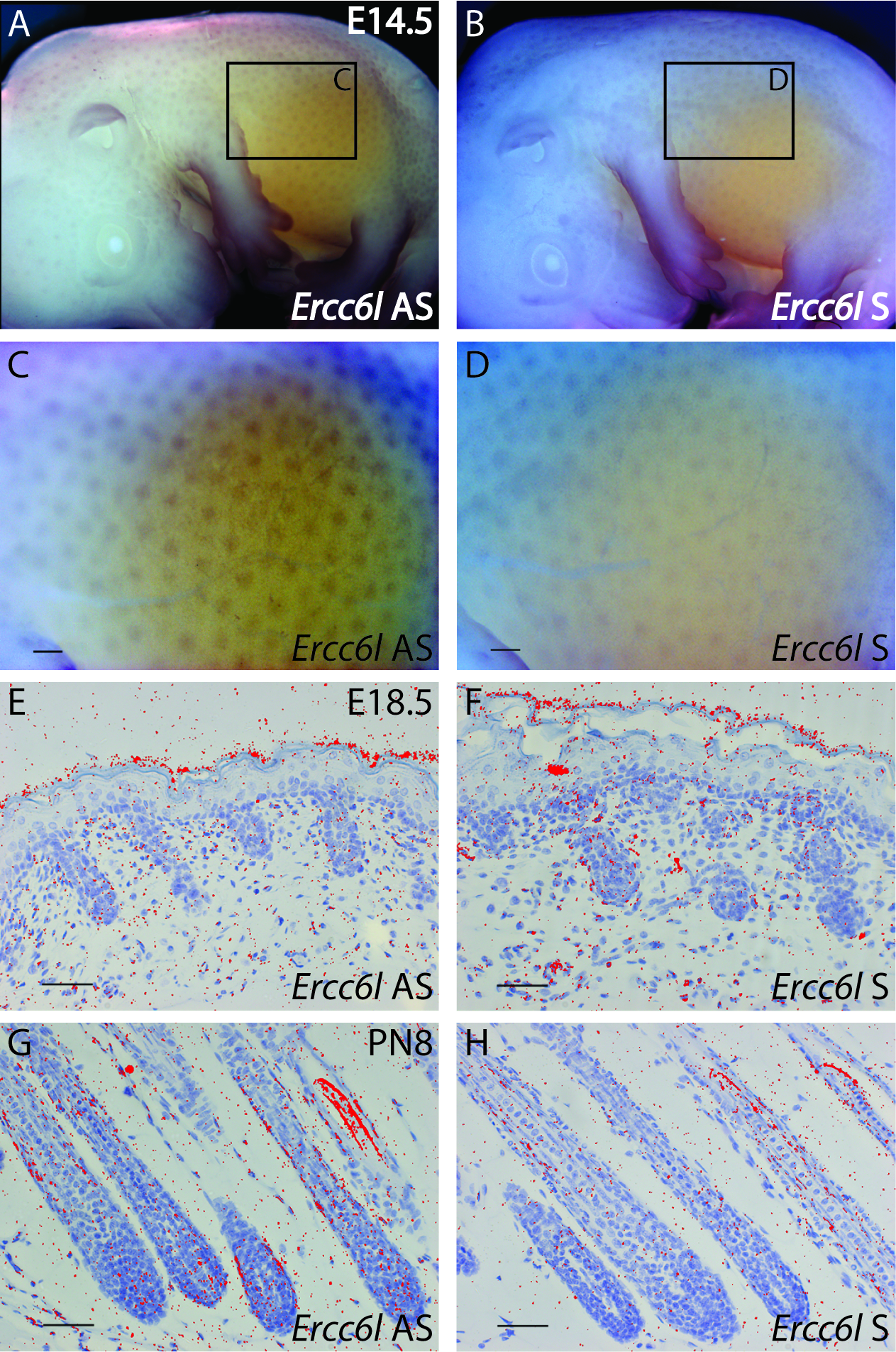

Supplement: S6 Fig — (A-F): Whole mount in situ hybridization of mouse embryos with dig-labelled Erccl6l antisense (AS) (A, C) and sense (S) (B, D) probes. No Ercc6l-specific signal was detected in the hair placodes at E14.5. C and D are close-ups of the inserts shown in A and B, respectively. (G-H): In situ hybridization with 35S-labeled Ercc6l AS (E, G) and S (F, H) probes during embryonic (E18.5) and postnatal (PN8) hair follicle morphogenesis. No specific signal was detected with more sensitive radioactive ISH technique, indicating the absence of Ercc6l transcripts in hair follicles at the stages analyzed. Scale bars are 200 μm. (TIF) [file pgen.1005427.s007.tif]
