# Supplementary material for: Hairless Streaks in Cattle Implicate TSR2 in Early Hair Follicle Formation
Source: PLoS Genet. 2015 Jul 23;11(7):e1005427. doi: 10.1371/journal.pgen.1005427 (PMC4512707; doi:10.1371/journal.pgen.1005427)
Supplement: S7 Fig — (A) Open reading frame (ORF) of both experimentally-detected wild type TSR2 transcripts and their encoded protein sequence. Sequences corresponding to exon 5 are shown in green. Note the underlined 9 nucleotides corresponding to exon 4b encoding three additional residues of TSR2 isoform 2. (B) Coding sequence and deduced protein sequence of two experimentally detected mutant TSR2 transcripts. The retained intron 4 sequence is shown in blue until a premature stop codon is reached. The utilization of an alternate AG splice site immediately downstream from the normal splice site, which is mutated, leads to a 7 nt shorter exon 5 (indicated by dots). The resultant mutant transcript 2 contains a frameshift which is predictive of a truncation. (PDF) [file pgen.1005427.s008.pdf]

A

ORF of the *TSR2* wild type transcript 1 (wt1; 576 nt)

ATGATGGCGGACGCTGCTGAAGATTCGCGAGCGCTCTTCGGGGCGGCAGTCCGTGCGGCGCTGGAGGCCTGGCCCGCCTTGCAGATCGCTGTGG
AGAACGGCTTCGGAGGTGTGTATAGCCAAGAGAAGGCTGAGTGGCTCGGGGGTGCAGTGGAGGAGTATTTCTTCCGCAATGCTGACTTGGAGCT
AGATGAGGTGGAGGACTTCCTCGGGGAGCTAATGATGAACGAGTTTGATACAGTTGTGGAGGATGGGAGTCTGCCCCAGGTGAGCCAGCAGCTG
CAGACCATGTTCCACCACTTCCAGAAGGGTGATAGGGCTGCTCTGAAGGAGATGGCCTCTCTCATCACCCAAAGAAAGTGCAAGGTCAGAGCCA
CTGCACTGCCGACAGCTGGAGAGACCGATGAGGATGATGATGCAAACAGCGTGGAGGAGATGGAGGTTGCAGCTACGAATGATGGGGCAGCTAC
AGATGGGGTCTGCCCCCAGCCTGAACCCTCTGGTCCAGACTCCCAGACTATTAAGGAAGAGGATATAGTGGAGGATGGCTGGACCATTGTCCGG
AGAAAGAAGTGA

TSR2 isoform 1 protein (191 aa)

MMADAAEDSRA**LFGAAVRAALEAWPALQIAVENGFGGVYSQEKAEWLGGAVEEYFFRNADLELDEVEDFLGELMMNEFDTVVEDGSLPQVSQQ**L
QTMFHHFQKGDRAALKEMASLITQRKCKVRATALPTAGETDEDDDANSVEEMEVAATNDGAATDGVCPQPEPSGPDSQTIKEEDIVEDGWTIVR
RKK

ORF of the *TSR2* wild type transcript 2 (wt2; 585 nt)

ATGATGGCGGACGCTGCTGAAGATTCGCGAGCGCTCTTCGGGGCGGCAGTCCGTGCGGCGCTGGAGGCCTGGCCCGCCTTGCAGATCGCTGTGG
AGAACGGCTTCGGAGGTGTGTATAGCCAAGAGAAGGCTGAGTGGCTCGGGGGTGCAGTGGAGGAGTATTTCTTCCGCAATGCTGACTTGGAGCT
AGATGAGGTGGAGGACTTCCTCGGGGAGCTAATGATGAACGAGTTTGATACAGTTGTGGAGGATGGGAGTCTGCCCCAGGTGAGCCAGCAGCTG
CAGACCATGTTCCACCACTTCCAGAAGGGTGATAGGGCTGCTCTGAAGGAGATGGCCTCTCTCATCACCCAAAGAAAGTGCAAGGTCAGAGCCA
CTGCACTGCCGACAGCTGGAGAGACCGATGAGGATGATGATGCAAACAGCGTGGAGGAGATGGAGGTGAAAGGGGTTGCAGCTACGAATGATGG
GGCAGCTACAGATGGGGTCTGCCCCCAGCCTGAACCCTCTGGTCCAGACTCCCAGACTATTAAGGAAGAGGATATAGTGGAGGATGGCTGGACC
ATTGTCCGGAGAAAGAAGTGA

TSR2 isoform 2 protein (194 aa)

MMADAAEDSRA**LFGAAVRAALEAWPALQIAVENGFGGVYSQEKAEWLGGAVEEYFFRNADLELDEVEDFLGELMMNEFDTVVEDGSLPQVSQQ**L
QTMFHHFQKGDRAALKEMASLITQRKCKVRATALPTAGETDEDDDANSVEEMEVKGVAATNDGAATDGVCPQPEPSGPDSQTIKEEDIVEDGWT
IVRRKK

B

ORF of the *TSR2* mutant transcript 1 (mt1; 477 nt)

ATGATGGCGGACGCTGCTGAAGATTCGCGAGCGCTCTTCGGGGCGGCAGTCCGTGCGGCGCTGGAGGCCTGGCCCGCCTTGCAGATCGCTGTGG
AGAACGGCTTCGGAGGTGTGTATAGCCAAGAGAAGGCTGAGTGGCTCGGGGGTGCAGTGGAGGAGTATTTCTTCCGCAATGCTGACTTGGAGCT
AGATGAGGTGGAGGACTTCCTCGGGGAGCTAATGATGAACGAGTTTGATACAGTTGTGGAGGATGGGAGTCTGCCCCAGGTGAGCCAGCAGCTG
CAGACCATGTTCCACCACTTCCAGAAGGGTGATAGGGCTGCTCTGAAGGAGATGGCCTCTCTCATCACCCAAAGAAAGTGCAAGGTCAGAGCCA
CTGCACTGCCGACAGCTGGAGAGACCGATGAGGATGATGATGCAAACAGCGTGGAGGAGATGGAG**gtgaaaggggtgcaactcttgggtgtgga**
gatgtag

Predicted mutant TSR2 protein 1 (157 aa)

MMADAAEDSRA**LFGAAVRAALEAWPALQIAVENGFGGVYSQEKAEWLGGAVEEYFFRNADLELDEVEDFLGELMMNEFTVVEDGSLPQVSQQ**LQ
TMFHHFQKGDRAALKEMASLITQRKCKVRATALPTAGETDEDDDANSVEEMEV**KGVQLLGVEM**

ORF of the *TSR2* mutant transcript 2 (mt2; 531 nt)

ATGATGGCGGACGCTGCTGAAGATTCGCGAGCGCTCTTCGGGGCGGCAGTCCGTGCGGCGCTGGAGGCCTGGCCCGCCTTGCAGATCGCTGTGG
AGAACGGCTTCGGAGGTGTGTATAGCCAAGAGAAGGCTGAGTGGCTCGGGGGTGCAGTGGAGGAGTATTTCTTCCGCAATGCTGACTTGGAGCT
AGATGAGGTGGAGGACTTCCTCGGGGAGCTAATGATGAACGAGTTTGATACAGTTGTGGAGGATGGGAGTCTGCCCCAGGTGAGCCAGCAGCTG
CAGACCATGTTCCACCACTTCCAGAAGGGTGATAGGGCTGCTCTGAAGGAGATGGCCTCTCTCATCACCCAAAGAAAGTGCAAGGTCAGAGCCA
CTGCACTGCCGACAGCTGGAGAGACCGATGAGGATGATGATGCAAACAGCGTGGAGGAGATGGAG.......CTACGAATGATGGGGCAGCTAC
AGATGGGGTCTGCCCCCAGCCTGAACCCTCTGGTCCAGACTCCCAGACTATTAAGGAAGAGGATATAG

Predicted mutant TSR2 protein 1 (176 aa)

MMADAAEDSRA**LFGAAVRAALEAWPALQIAVENGFGGVYSQEKAEWLGGAVEEYFFRNADLELDEVEDFLGELMMNEFDTVVEDGSLPQVSQQ**L
QTMFHHFQKGDRAALKEMASLITQRKCKVRATALPTAGETDEDDDANSVEEMELRMMGQLQMGSAPSLNPLVQTPRLLRKRI
