# Supplementary material for: Hairless Streaks in Cattle Implicate TSR2 in Early Hair Follicle Formation
Source: PLoS Genet. 2015 Jul 23;11(7):e1005427. doi: 10.1371/journal.pgen.1005427 (PMC4512707; doi:10.1371/journal.pgen.1005427)
Supplement: S1 Table — (PDF) [file pgen.1005427.s009.pdf]

S1 Table List of filtered sequence variants.

(light blue: these 19 coding variants were present among the data of the 1000 bull genome project;
dark blue: these 29 variants (including 2 coding) were private to the sequenced cow with streaked hairlessness)

CHR	POSITION	REF	ALT	GENE	TRANSCRIPT_ID	CODON	AMINO ACID EXCHANGE	EFFECT	IMPACT	FUNCLASS
7	82984104	G	T					INTERGENIC	MODIFIER	
7	87121767	A	C					INTERGENIC	MODIFIER	
7	88347918	G	T					INTERGENIC	MODIFIER	
7	92679100	G	A	GPR98	ENSBTAT00000007926	Gag/Aag	E4734K	NON_SYNONYMOUS_CODING	MODERATE	MISSENSE
7	94663554	G	A					INTERGENIC	MODIFIER	
7	98191740	T	G	PCSK1	ENSBTAT00000027775	753		INTRON	MODIFIER	
7	101312678	A	G					INTERGENIC	MODIFIER	
7	102678037	C	A					INTERGENIC	MODIFIER	
7	102678057	A	T					INTERGENIC	MODIFIER	
7	102697724	A	G					INTERGENIC	MODIFIER	
7	102730561	G	T					INTERGENIC	MODIFIER	
7	102745774	G	A					INTERGENIC	MODIFIER	
7	102882724	A	G					INTERGENIC	MODIFIER	
7	102925636	T	C					INTERGENIC	MODIFIER	
7	102942242	C	T					INTERGENIC	MODIFIER	
7	102994911	C	T					INTERGENIC	MODIFIER	
7	103081418	C	T					INTERGENIC	MODIFIER	
7	103275452	G	A					INTERGENIC	MODIFIER	
7	103902577	G	A	SLCO6A1	ENSBTAT00000042642	726		INTRON	MODIFIER	
7	104866194	C	T					INTERGENIC	MODIFIER	
7	105455521	A	G					INTERGENIC	MODIFIER	
7	106075845	T	A					INTERGENIC	MODIFIER	
7	107067970	A	G					INTERGENIC	MODIFIER	
7	108174799	G	A					INTERGENIC	MODIFIER	
7	108227569	C	T					INTERGENIC	MODIFIER	
7	111565794	G	A	MAN2A1	ENSBTAT00000042616	1145		INTRON	MODIFIER	
7	111600171	T	C	MAN2A1	ENSBTAT00000042616	1145		INTRON	MODIFIER	
7	112594848	G	A					INTERGENIC	MODIFIER	
X	55177392	G	A	Q3ZC95_BOVIN	ENSBTAT00000030136	Cgc/Tgc	R490C	NON_SYNONYMOUS_CODING	MODERATE	MISSENSE
X	56796373	C	T	ENSBTAG00000046314	ENSBTAT00000046511	Gtc/Atc	V363I	NON_SYNONYMOUS_CODING	MODERATE	MISSENSE
X	59849575	C	A	FRMPD3	ENSBTAT00000004058	gaG/gaT	E1129D	NON_SYNONYMOUS_CODING	MODERATE	MISSENSE
X	59915923	C	T	FRMPD3	ENSBTAT00000004058	Gat/Aat	D11N	NON_SYNONYMOUS_CODING	MODERATE	MISSENSE
X	60948964	T	C	VSIG1	ENSBTAT00000010335	ccT/ccC	P122	SYNONYMOUS_CODING	LOW	SILENT
X	71066549	G	A	BRWD3	ENSBTAT00000021597	cGa/cAa	R1096Q	NON_SYNONYMOUS_CODING	MODERATE	MISSENSE
X	83572401	G	A	ERC6L_BOVIN	ENSBTAT00000007362	Gct/Act	A18T	NON_SYNONYMOUS_CODING	MODERATE	MISSENSE
X	85111232	G	A	TEX11	ENSBTAT00000021646	cGt/cAt	R264H	NON_SYNONYMOUS_CODING	MODERATE	MISSENSE
X	92095101	C	T	PQBP1_BOVIN	ENSBTAT00000024621	Cca/Tca	P50S	NON_SYNONYMOUS_CODING	MODERATE	MISSENSE
X	92370868	G	A	FOXP3	ENSBTAT00000017660	ttC/ttT	F340	SYNONYMOUS_CODING	LOW	SILENT
X	96448123	G	A	HUWE1	ENSBTAT00000008060			SPLICE_SITE_REGION	LOW	
X	97363937	A	G	TSR2_BOVIN	ENSBTAT00000021080			SPLICE_SITE_ACCEPTOR	HIGH	
X	99530471	A	G	ENSBTAG00000045616	ENSBTAT00000064453	tAc/tGc	Y163C	NON_SYNONYMOUS_CODING	MODERATE	MISSENSE
X	100431354	G	T	ZC3H12B	ENSBTAT00000002858	ggC/ggA	G21	SYNONYMOUS_CODING	LOW	SILENT
X	111387588	G	T	ENSBTAG00000045660	ENSBTAT00000064739	Ctc/Atc	L42I	NON_SYNONYMOUS_CODING	MODERATE	MISSENSE
X	119318161	G	A	ENSBTAG00000037496	ENSBTAT00000053241	aCc/aTc	T241I	NON_SYNONYMOUS_CODING	MODERATE	MISSENSE
X	132099758	G	A	CDKL5	ENSBTAT00000009766	aCc/aTc	T567I	NON_SYNONYMOUS_CODING	MODERATE	MISSENSE
X	133640136	C	T	REPS2	ENSBTAT00000016315	ccG/ccA	P302	SYNONYMOUS_CODING	LOW	SILENT
X	141157459	G	A	FRMPD4	ENSBTAT00000023650	agC/agT	S907	SYNONYMOUS_CODING	LOW	SILENT
X	143430122	A	C	ENSBTAG00000002158	ENSBTAT00000056084	gcT/gcG	A31	SYNONYMOUS_CODING	LOW	SILENT
